# Regulation of Neuroinflammation by Microglial DUBA‐IRAK1‐IKKβ Signaling Loop

**DOI:** 10.1002/advs.202503972

**Published:** 2025-08-04

**Authors:** Zhenhu Zhu, Zhongding Li, Kate Lykke Lambertsen, Zijun Cao, Meng Chen, Yanqi Xu, Bettina Hjelm Clausen, Keshuo Jin, Yiting Lu, Fuqi Mei, Xue Du, Kangmin Chen, Huiyuan Bai, Xian Su, Bincheng Zhou, Baohua Liu, Ran Li, Chaoqun Wang, Yuhua Li, Xiang Cai, Dirk Schlüter, Weihong Song, Xu Wang

**Affiliations:** ^1^ School of Pharmaceutical Sciences Wenzhou Medical University Wenzhou 325035 China; ^2^ Oujiang Laboratory (Zhejiang Lab for Regenerative Medicine Vision and Brain Health) Wenzhou 325000 China; ^3^ Department of Neurobiology Research Institute of Molecular Medicine University of Southern Denmark Odense 5230 Denmark; ^4^ BRIDGE ‐ Brain Research – Inter Disciplinary Guided Excellence Department of Clinical Research University of Southern Denmark Odense 5230 Denmark; ^5^ Department of Neurology Odense University Hospital Odense 5000 Denmark; ^6^ Department of Neurological Rehabilitation The Second Affiliated Hospital and Yuying Children's Hospital of Wenzhou Medical University Wenzhou 325027 China; ^7^ Key Laboratory of Alzheimer's Disease of Zhejiang Province Zhejiang Provincial Clinical Research for Mental Disorders Center for Geriatric Medicine and Institute of Aging The First‐affiliated Hospital Wenzhou Medical University Wenzhou Zhejiang 325035 China; ^8^ College of Life Sciences Northeast Forestry University Harbin 150040 China; ^9^ Institute of Medical Microbiology and Hospital Epidemiology Hannover Medical School 30625 Hannover Germany

**Keywords:** DUBA, microglia, neuroinflammation, signal transduction, ubiquitination

## Abstract

Activation of microglia is closely associated with neuroinflammation. However, the cell‐intrinsic molecular mechanisms translating microglia activation into neuroinflammation are only partially understood. Here, it is shown that deubiquitinating enzyme A (DUBA) is upregulated in microglia under neuroinflammatory conditions in both mice and humans. Mechanistically, activation of microglia induces DUBA self‐deubiquitination and stabilization, leading to the rapid upregulation of DUBA protein levels. In turn, stabilized DUBA increases proinflammatory gene induction in activated microglia by enhancing the activation of nuclear factor‐κB (NF‐κB) and mitogen‐activated protein kinase (MAPK) signaling. Of note, DUBA promotes NF‐κB and MAPK activation by stabilizing interleukin‐1 receptor activated kinase 1 (IRAK1) through K48 deubiquitination. Importantly, specific ablation of DUBA in microglia mitigates lipopolysaccharide‐induced depression‐like behavior and ischemic stroke injury in mice by limiting neuroinflammation. Collectively, these findings establish DUBA as a key regulator of microglia in neuroinflammation and uncover novel molecular mechanisms for DUBA in inflammatory signal transduction.

## Graphical abstract

The protein levels of deubiquitinating enzyme A (DUBA) are rapidly upregulated in microglia after activation of Toll‐like receptor 4 (TLR4). In turn, DUBA potentiates TLR4‐induced proinflammatory signal transduction in microglia by stabilizing interleukin‐1 receptor activated kinase 1 (IRAK1) through K48 deubiquitination. Targeted ablation of microglial DUBA attenuates lipopolysaccharide‐induced depression‐like behavior and ischemic stroke injury in mice by reducing neuroinflammation.

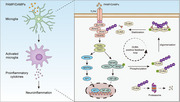

## 1 Introduction

Microglia actively participate in CNS development, homeostasis, and diseases.^[^ ^1^ ^]^ Microglia sense tissue damage and microbial invasion by recognizing damage‐associated molecular patterns (DAMPs) and pathogen‐associated molecular patterns (PAMPs), respectively, through pattern recognition receptors (PRRs) and subsequently produce a plethora of inflammatory molecules, leading to the establishment of a neuroinflammatory milieu.^[^ ^2^ ^]^ In particular, stimulation of the PRR Toll‐like receptor 4 (TLR4) by lipopolysaccharide (LPS), a PAMP derived from gram‐negative bacteria, induces the strong activation of microglia. Neuroinflammation is a common pathological hallmark of various CNS diseases such as stroke, neurodegenerative diseases, CNS autoimmune diseases, and CNS infections.^[^ ^3^ ^]^

Upon PRR activation by DAMPs or PAMPs, multiple intracellular signaling pathways, such as nuclear factor‐κB (NF‐κB) and mitogen‐activated protein kinase (MAPK) pathways, are activated to drive microglial activation.^[^ ^4^ ^]^ Signal transduction is tightly regulated by post‐translational modifications (PTMs) including ubiquitination.^[^ ^5^ ^]^ Ubiquitination, catalyzed sequentially by E1, E2, and E3 ubiquitinating enzymes, involves the covalent attachment of one or more 76‐amino‐acid ubiquitin molecules to the substrate protein.^[^ ^6^ ^]^ As a counter‐regulating mechanism, deubiquitinating enzymes (DUBs) reverse ubiquitination by removing ubiquitin molecules.^[^ ^7^ ^]^ Ubiquitin‐modifying enzymes critically regulate microglial activation in neuroinflammation, and dysregulation of DUBs has been implicated in neurological disorders.^[^ ^8^ ^]^ For example, autoimmune CNS inflammation is aggravated by the E3 ligase Pelino 1, which enhances TLR4 signaling in microglia by promoting the ubiquitination‐dependent degradation of TNF receptor‐associated factor 3 (TRAF3).^[^ ^9^ ^]^ In sharp contrast, the DUBs USP18 and A20 ameliorate autoimmune CNS inflammation by inhibiting microglial activation.^[^ ^10^ ^]^ Moreover, the DUB USP25 affects ischemic stroke injury and Alzheimer's Disease by regulating microglia‐associated neuroinflammation.^[^ ^11^ ^]^

Deubiquitinating enzyme A (DUBA) is a DUB that has been implicated in the regulation of various inflammatory signaling pathways. Initially, DUBA was found to inhibit PRR‐induced type I interferon production.^[^ ^12^ ^]^ In addition, DUBA inhibits interleukin (IL)‐17 production in T‐helper type 17 (Th17) cells and thereby attenuates Th17 cell‐mediated intestinal inflammation.^[^ ^13^ ^]^ Recent studies demonstrate that DUBA controls inflammatory responses by regulating PRR‐induced NF‐κB and MAPK signaling pathways in innate immune cells.^[^ ^14^ ^]^ However, whether DUBA participates in microglial activation and neuroinflammation remains elusive.

In the present study, we discovered that TLR4 activation induced the accumulation of DUBA in microglia through self‐deubiquitination. In turn, DUBA enhanced TLR4 signaling by stabilizing IL‐1 receptor activated kinase 1 (IRAK1) through K48 deubiquitination. Specific ablation of microglial DUBA significantly ameliorated neuroinflammation upon challenge with LPS or ischemic stroke in mice. Together, this study demonstrates a crucial role for DUBA in microglial activation and neuroinflammation, and provides a novel regulatory mechanism for TLR signaling.

## 2 Results

### 2.1 LPS Upregulates DUBA Expression

**Figure 1:**
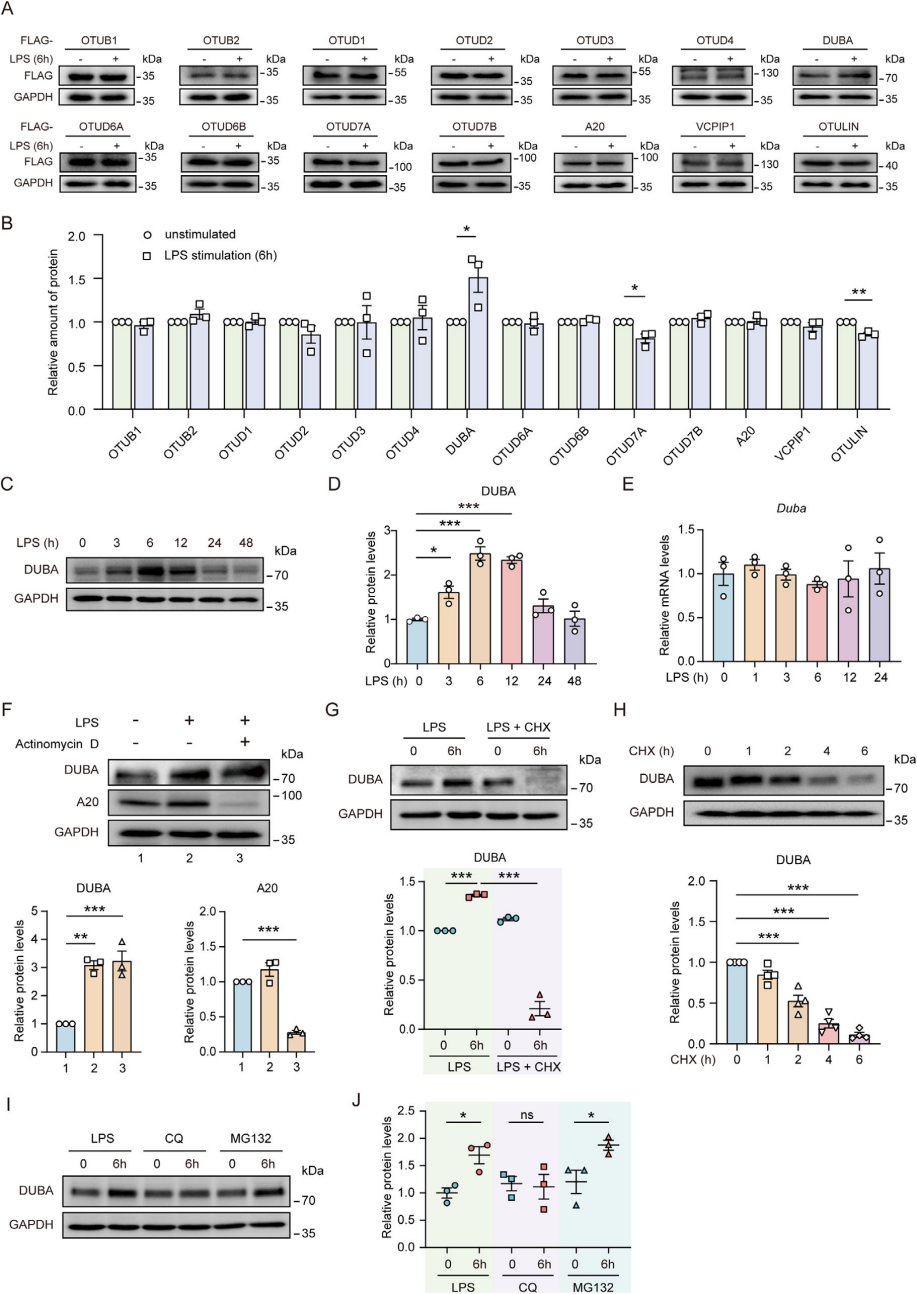
DUBA expression is induced by LPS stimulation. A,B) BV2 cells were transfected with indicated plasmids for 24 h, followed by LPS (500 ng mL^−1^) stimulation for 6 h. Whole‐cell lysates were analyzed by Western blot. Representative immunoblots A) and quantification B) are shown (n = 3). Mean ± SEM. * *p* < 0.05. C,D) BV2 cells were stimulated with LPS (500 ng mL^−1^) for indicated periods of time, followed by Western blot analysis. Representative immunoblots C) and quantification D) are shown (n = 3). Mean ± SEM. * *p* < 0.05, *** *p* < 0.001. E) BV2 cells were stimulated with LPS (500 ng mL^−1^) for indicated periods of time. Transcription of *Duba* mRNA was determined by qRT‐PCR (n = 3). Mean ± SEM. F) BV2 cells were left untreated or stimulated with LPS (500 ng mL^−1^) in the absence or presence of Actinomycin D (1 µM) for 6 h. Whole‐cell lysates were analyzed by Western blot. Representative immunoblots (upper panel) and quantification (lower panel) are shown (n = 3). Mean ± SEM. ** *p* < 0.01. *** *p* < 0.001. G) BV2 cells were stimulated with LPS (500 ng mL^−1^) in the presence or absence of cycloheximide (CHX; 10 µM) for indicated periods of time. Whole‐cell lysates were analyzed by Western blot. Representative immunoblots (upper panel) and quantification (lower panel) are shown (n = 3). Mean ± SEM. *** *p* < 0.001. H) BV2 cells were stimulated with CHX (10 µM) for indicated periods of time. Whole‐cell lysates were analyzed by Western blot. Representative immunoblots (upper panel) and quantification (lower panel) are shown (n = 4). Mean ± SEM. *** *p* < 0.001. I,J) BV2 cells were stimulated with LPS (500 ng mL^−1^), CQ (10 µM), or MG132 (5 µM) for indicated periods of time, followed by Western blot analysis. Representative immunoblots (I) and quantification (J) are shown (n = 3). Mean ± SEM. * *p* < 0.05.

To identify potential regulators of TLR4 signaling in microglia, we overexpressed FLAG‐tagged DUBs of the ovarian tumor protease (OTU) family in BV2 cells and then stimulated them with LPS for 6 hours. The short‐term LPS stimulation rapidly upregulated DUBA and downregulated OTUD7A and OTULIN, with DUBA being the most significantly changed DUB (**Figure** **1**A,B). We then verified the screening result by analyzing endogenous DUBA expression after LPS stimulation and found that LPS rapidly increased DUBA protein levels in a time‐dependent manner (Figure 1C,D). In contrast, the mRNA levels of *Duba* were not altered by LPS stimulation (Figure 1E), indicating that the LPS‐induced upregulation of DUBA is transcription‐independent. The transcription inhibitor actinomycin D strongly inhibited LPS‐induced upregulation of A20, which is dependent on *de novo* gene transcription (Figure 1F). However, actinomycin D failed to inhibit LPS‐induced upregulation of DUBA (Figure 1F), showing that LPS induced DUBA expression in a transcription‐independent way. In sharp contrast to actinomycin D, the protein synthesis inhibitor cycloheximide (CHX) strongly reduced DUBA protein levels (Figure 1G), indicating that DUBA protein levels may be tightly regulated by synthesis and degradation. Indeed, the CHX chase assay revealed that DUBA protein could be rapidly degraded (Figure 1H). Proteasomes and lysosomes are two major machineries mediating proteolysis.^[^ ^8^ ^]^ Treatment with the proteasome inhibitor MG132, rather than the lysosome inhibitor chloroquine (CQ), increased DUBA protein levels in BV2 cells (Figure 1I,J), suggesting that DUBA is predominantly degraded through the proteasome. Moreover, MG132 treatment increased DUBA expression as efficiently as LPS stimulation (Figure 1I,J), indicating that LPS may upregulate DUBA protein levels by inhibiting the ubiquitin‐proteasome system (UPS).

### 2.2 LPS Inhibits the Degradation of DUBA by Inducing its Deubiquitination

**Figure 2:**
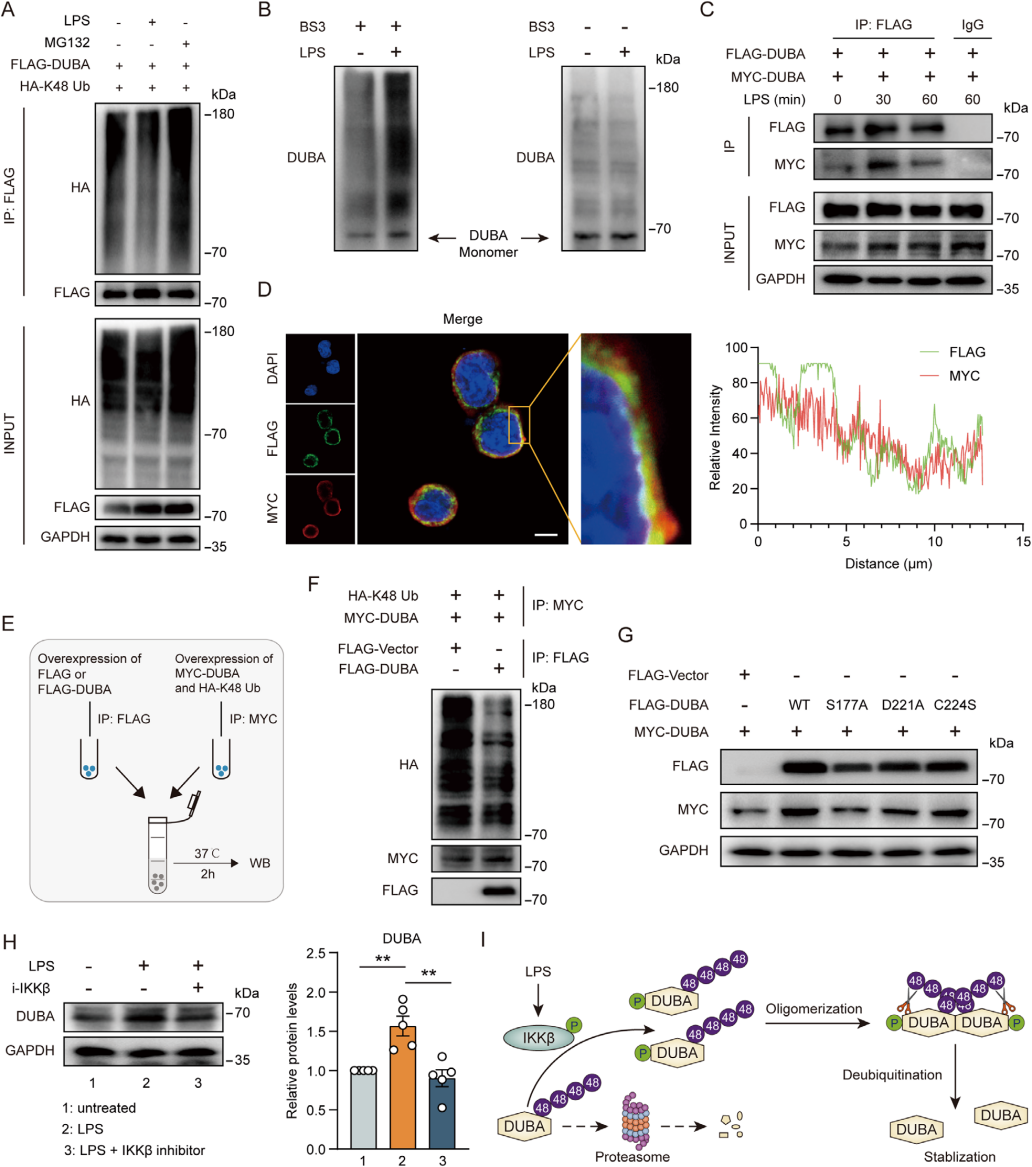
LPS inhibits proteasome degradation by regulating the formation of DUBA oligomers. A) BV2 cells were transfected with FLAG‐DUBA and HA‐K48 Ub plasmids for 24 h, followed by stimulation with LPS (500 ng mL^−1^) or MG132 (5 µM) for 6 h. Proteins were immunoprecipitated from whole‐cell lysates with anti‐FLAG antibodies and then analyzed by Western blot. B) BV2 cells were left untreated or stimulated with LPS (500 ng mL^−1^) for 30 min before lysis. Whole‐cell lysates were treated with or without BS3 (3 mM) and then analyzed by Western blot. C) BV2 cells were transfected with FLAG‐DUBA and MYC‐DUBA plasmids for 24 h, followed by stimulation with LPS (500 ng mL^−1^) for indicated periods of time. Proteins were immunoprecipitated from whole‐cell lysates and then analyzed by Western blot. D) BV2 cells were transfected with FLAG‐DUBA and MYC‐DUBA plasmids for 24 h. The subcellular distribution of indicated proteins was determined by immunofluorescence. Scale bar, 10 µm. E,F) Schematic diagram (E) and representative immunoblots (F) of the in vitro deubiquitination assay. G) BV2 cells were transfected with indicated plasmids for 24 h. Whole‐cell lysates were analyzed by Western blot. H) BV2 cells were left untreated or stimulated with LPS (500 ng mL^−1^) in the absence or presence of IKK‐β inhibitor (10 µM) for 6 h. Whole‐cell lysates were analyzed by Western blot. Representative immunoblots (left panel) and DUBA quantification (right panel) are shown. Mean ± SEM. ** *P* < 0.01. I) Schematic diagram of LPS‐induced DUBA stabilization.

Given that proteins degraded in the proteasome are normally labeled with K48‐linked polyubiquitin chains, we next analyzed the effect of LPS on DUBA ubiquitination. LPS stimulation markedly reduced K48‐specific ubiquitination of DUBA (**Figure** **2**A). We found that DUBA could form oligomers, which was strongly promoted by LPS stimulation (Figure 2B). Both immunoprecipitation and immunofluorescence results confirmed that FLAG‐tagged DUBA interacted with MYC‐tagged DUBA in cells (Figure 2C,D; Figure S1A, Supporting Information). Considering that DUBA is a DUB, we hypothesized that DUBA aggregated upon LPS stimulation and then cleaved K48 polyubiquitin chains from each other. To confirm this hypothesis, an in vitro deubiquitination assay was performed (Figure 2E). Indeed, FLAG‐tagged DUBA could efficiently remove K48 polyubiquitin chains established on MYC‐tagged DUBA (Figure 2F). Consistently, overexpression of FLAG‐DUBA enhanced MYC‐DUBA expression in BV2 cells (Figure 2G; Figure S1B, Supporting Information). These data suggested that DUBA is self‐deubiquitinated. Inactive mutation of D221 and C224 partially reduced expression levels of FLAG‐DUBA and MYC‐DUBA (Figure 2G; Figure S1B, Supporting Information), indicating that both D221 and C224 residues are required for the self‐deubiquitination of DUBA.

A previous report showed that the DUB activity of DUBA is dependent on S177 phosphorylation.^[^ ^15^ ^]^ We also found that inactive mutation of S177 decreased the expression of FLAG‐DUBA and completely failed to increase MYC‐DUBA expression (Figure 2G; Figure S1B, Supporting Information). Given the importance of S177 phosphorylation for DUBA activity, we proceeded to identify the kinase that phosphorylated DUBA. Inhibition of the inhibitor of κB kinases (IKKs), rather than MAPKs, eliminated the upregulation of DUBA after LPS stimulation (Figure S2A,B, Supporting Information). Among IKKs, IKKβ could interact with DUBA (Figure S3A, Supporting Information). Furthermore, specific inhibition of IKKβ abolished the effect of LPS on DUBA expression (Figure 2H), showing that LPS induced DUBA phosphorylation through IKKβ. Collectively, these results demonstrate that LPS induces the phosphorylation and activation of DUBA through IKKβ and thereby promoted DUBA oligomerization and self‐deubiquitination, leading to its stabilization and upregulation (Figure 2I).

### 2.3 DUBA Promotes NF‐κB and MAPK‐Mediated Inflammatory Responses by Increasing IRAK1 Expression in Microglia

**Figure 3:**
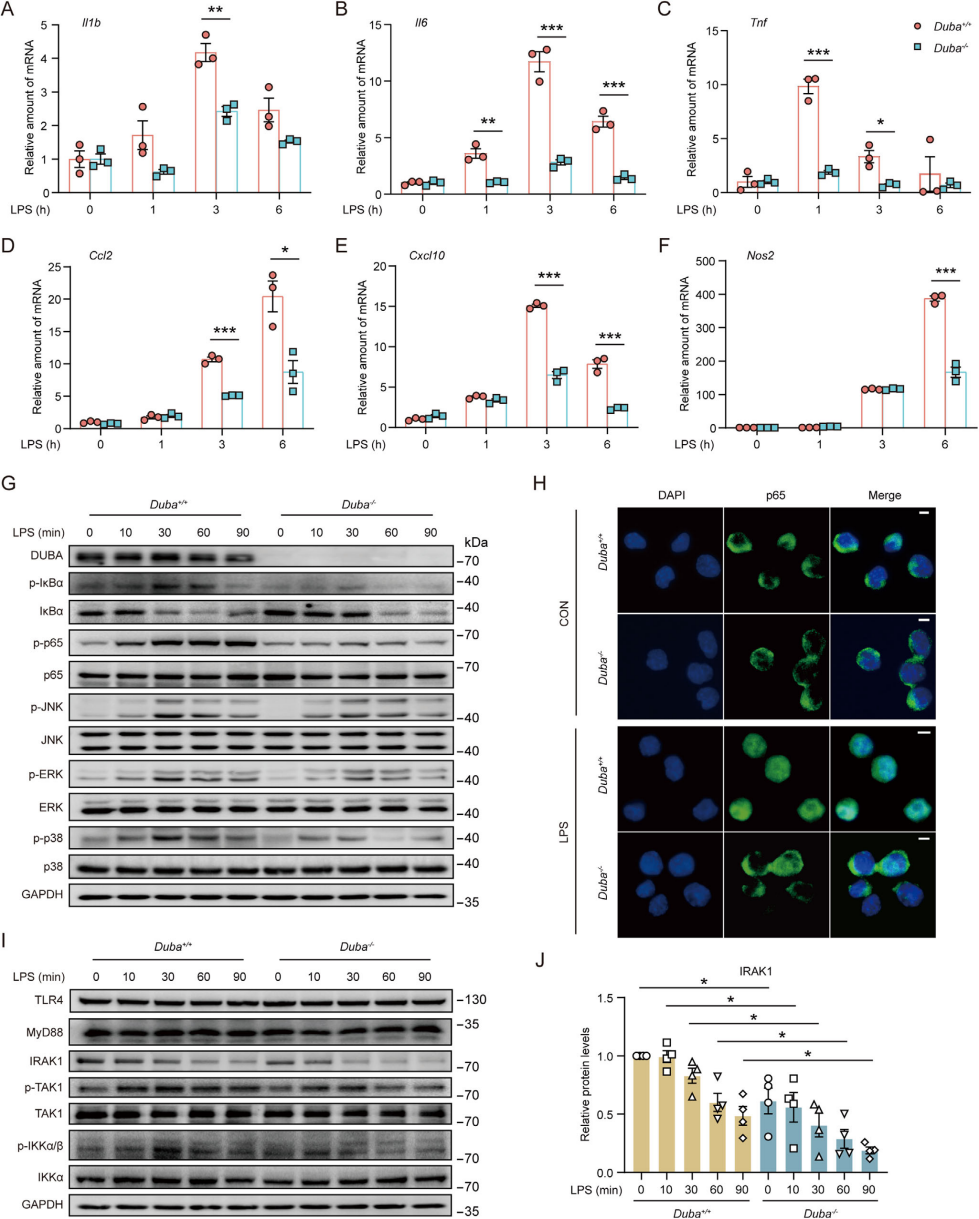
Knockdown of DUBA diminishes LPS‐induced proinflammatory gene transcription and signal transduction in microglia. A‐F) *Duba* ^+/+^ and *Duba* ^‐/‐^ BV2 cells were stimulated with LPS (500 ng mL^−1^) for indicated periods of time. The transcription of *Il1b* (A), *Il6* (B), *Tnf* (C), *Ccl2* (D), *Cxcl10* (E), and *Nos2* (F) was determined by qRT‐PCR (n = 3). Mean ± SEM. *P < 0.05, **P < 0.01, ***P < 0.001. G) *Duba* ^+/+^ and *Duba* ^‐/‐^ BV2 cells were stimulated with LPS (500 ng mL^−1^) for indicated periods of time. Whole‐cell lysates were analyzed by Western blot. H) Representative immunofluorescence staining of p65 in *Duba* ^+/+^ and *Duba* ^‐/‐^ BV2 cells treated with LPS (500 ng mL^−1^) for 0 and 30 min. Scale bar, 10 µm. I) Western blot was performed to determine protein expression in *Duba* ^+/+^ and *Duba* ^‐/‐^ BV2 cells stimulated with LPS (500 ng mL^−1^). J) Relative quantification of IRAK1 protein levels in *Duba* ^+/+^ and *Duba* ^‐/‐^ BV2 cells (n = 4). Mean ± SEM. *P < 0.05.

The rapid activation and stabilization of DUBA after LPS stimulation implies a possible role of DUBA in LPS‐induced inflammatory responses. To explore the function of DUBA in microglia, we generated *Duba* ^‐/‐^ BV2 cells using CRISPR/Cas9 gene editing and found that DUBA deficiency significantly decreased the production of proinflammatory molecules in BV2 cells upon LPS stimulation (**Figure** **3**A ‐ F). We found that DUBA deficiency reduced the activation of NF‐κB and MAPK signaling pathways in response to LPS (Figure 3G; Figure S4A‐F, Supporting Information). Consistently, LPS‐induced nuclear translocation of p65 was also impaired in the absence of DUBA (Figure 3H), showing that DUBA promoted LPS‐induced inflammatory responses by enhancing NF‐κB and MAPK signaling.

To decipher the underlying molecular mechanism, we analyzed the effect of DUBA on proximal signaling molecules of the TLR4 signaling. MyD88 is an upstream organizing center of the TLR4 signaling, however, neither the protein abundance nor the oligomerization of MyD88 was affected by DUBA deficiency in LPS‐stimulated BV2 cells (Figure 3I; Figures S4G ‐ I and S5, Supporting Information). IRAK1 is a pivotal upstream signaling molecule that is recruited to the MyD88 complex upon TLR4 activation and critically mediates downstream signal transduction.^[^ ^16^ ^]^ Interestingly, DUBA ablation significantly reduced the protein levels of IRAK1 in BV2 cells (Figure 3I,J). Together, these findings demonstrate that DUBA enhances LPS‐induced NF‐κB and MAPK signaling by increasing IRAK1 protein levels, thereby promoting the production of proinflammatory molecules in microglia.

### 2.4 DUBA Interacts with IRAK1 and Reduces IRAK1 Degradation

**Figure 4:**
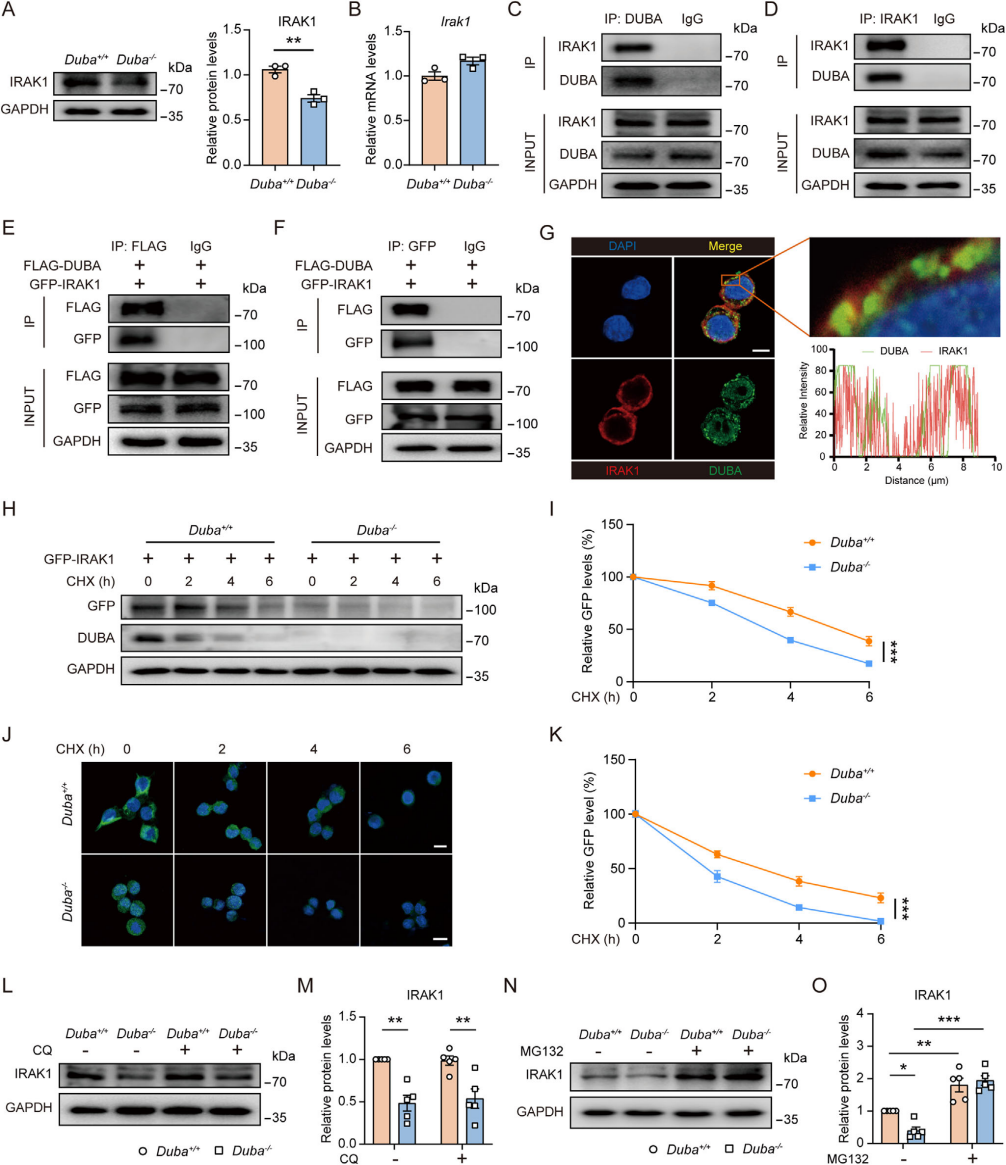
DUBA inhibits the proteasomal degradation of IRAK1. A) Western blot was performed to determine IRAK1 protein levels in *Duba* ^+/+^ and *Duba* ^‐/‐^ BV2 cells. Representative immunoblots (left panel) and quantification (right panel) are shown (n = 3). Mean ± SEM. ** *P* < 0.01. B) Transcription of *Irak1* mRNA in *Duba* ^+/+^ and *Duba* ^‐/‐^ BV2 cells was determined by qRT‐PCR (n = 3). Mean ± SEM. C,D) BV2 cell lysates were subjected to immunoprecipitation with antibodies against DUBA (C) or IRAK1 (D), followed by Western blot analysis. E,F) BV2 cells were transfected with FLAG‐DUBA and GFP‐IRAK1 plasmids for 24 h. Whole‐cell lysates were subjected to immunoprecipitation with antibodies against FLAG (E) or GFP (F), followed by Western blot analysis. G) The subcellular distribution of DUBA and IRAK1 was determined by immunofluorescence. Scale bar, 10 µm. H,I) BV2 cells were transfected with GFP‐IRAK1 plasmids for 24 h, followed by treatment with CHX (10 µM) for indicated periods of time. Whole‐cell lysates were analyzed by Western blot. Representative immunoblots (H) and quantification (I) are shown (n = 3). Mean ± SEM. ***p < 0.001. J,K) After transfection with GFP‐IRAK1 plasmids for 24 h, *Duba* ^+/+^ and *Duba* ^‐/‐^ BV2 cells were treated with CHX (10 µM) for indicated periods of time. GFP‐IRAK1 protein abundance was analyzed by immunofluorescence. Representative immunofluorescence staining (J) and quantification (K) are shown (n = 3). Mean ± SEM. ***P < 0.001. Scale bar, 20 µm. L,M) *Duba* ^+/+^ and *Duba* ^‐/‐^ BV2 cells were treated with 10 µM CQ for 6 h or left untreated. Whole‐cell lysates were analyzed by Western blot. Representative immunoblots (L) and quantification (M) are shown (n = 5). Mean ± SEM. **p < 0.01. N,O) *Duba* ^+/+^ and *Duba* ^‐/‐^ BV2 cells were treated with 5 µM MG132 for 6 h or left untreated. Whole‐cell lysates were analyzed by Western blot. Representative immunoblots (N) and quantification (O) are shown (n = 5). Mean ± SEM. *p < 0.05, **p < 0.01, ***p < 0.001.

Further analysis confirmed that knockout or knockdown of DUBA significantly reduced protein levels of IRAK1 in BV2 cells (**Figure** **4**A; Figure S6A,B, Supporting Information). Consistently, overexpression of DUBA increased IRAK1 protein levels in BV2 cells (Figure S6C, Supporting Information). Moreover, ectopic expression of DUBA increased protein levels of IRAK1 in both *Duba* ^+/+^ and *Duba* ^‐/‐^ BV2 cells and blunted the difference in IRAK1 expression between the two genotypes (Figure S6D, Supporting Information). Of note, DUBA deletion did not reduce *Irak1* transcription (Figure 4B), suggesting that DUBA does not affect the *de novo* synthesis of IRAK1. Given that IRAK1 is degraded after ubiquitination and that DUBA stabilizes protein substrate by removing ubiquitin modification,^[^ ^13^, ^17^ ^]^ DUBA may increase IRAK1 protein levels by inhibiting its degradation. To confirm this hypothesis, we first checked whether DUBA directly interacted with IRAK1. Immunoprecipitation results showed that DUBA interacted with IRAK1 at both endogenous and exogenous levels (Figure 4C–F). However, DUBA could not interact with IRAK2, IRAK4, TRAF6, TAK1, or TAB2 (Figure S3A,B, Supporting Information). Moreover, immunofluorescence staining revealed that IRAK1 and DUBA co‐localized in the cytoplasm (Figure 4G). Functionally, DUBA deficiency significantly accelerated the degradation of IRAK1, as revealed by Western blot and immunofluorescence (Figure 4H–K). Furthermore, inhibition of proteasomes, rather than lysosomes, increased IRAK1 protein abundance to a similar level in *Duba* ^+/+^ and *Duba* ^‐/‐^ BV2 cells (Figure 4L–O), indicating that DUBA predominantly inhibits the proteasomal degradation of IRAK1. Together, these results show that DUBA interacts with IRAK1 and increases the protein abundance of IRAK1 by inhibiting its degradation.

### 2.5 DUBA Removes K48‐Linked Polyubiquitin Chains from IRAK1

**Figure 5:**
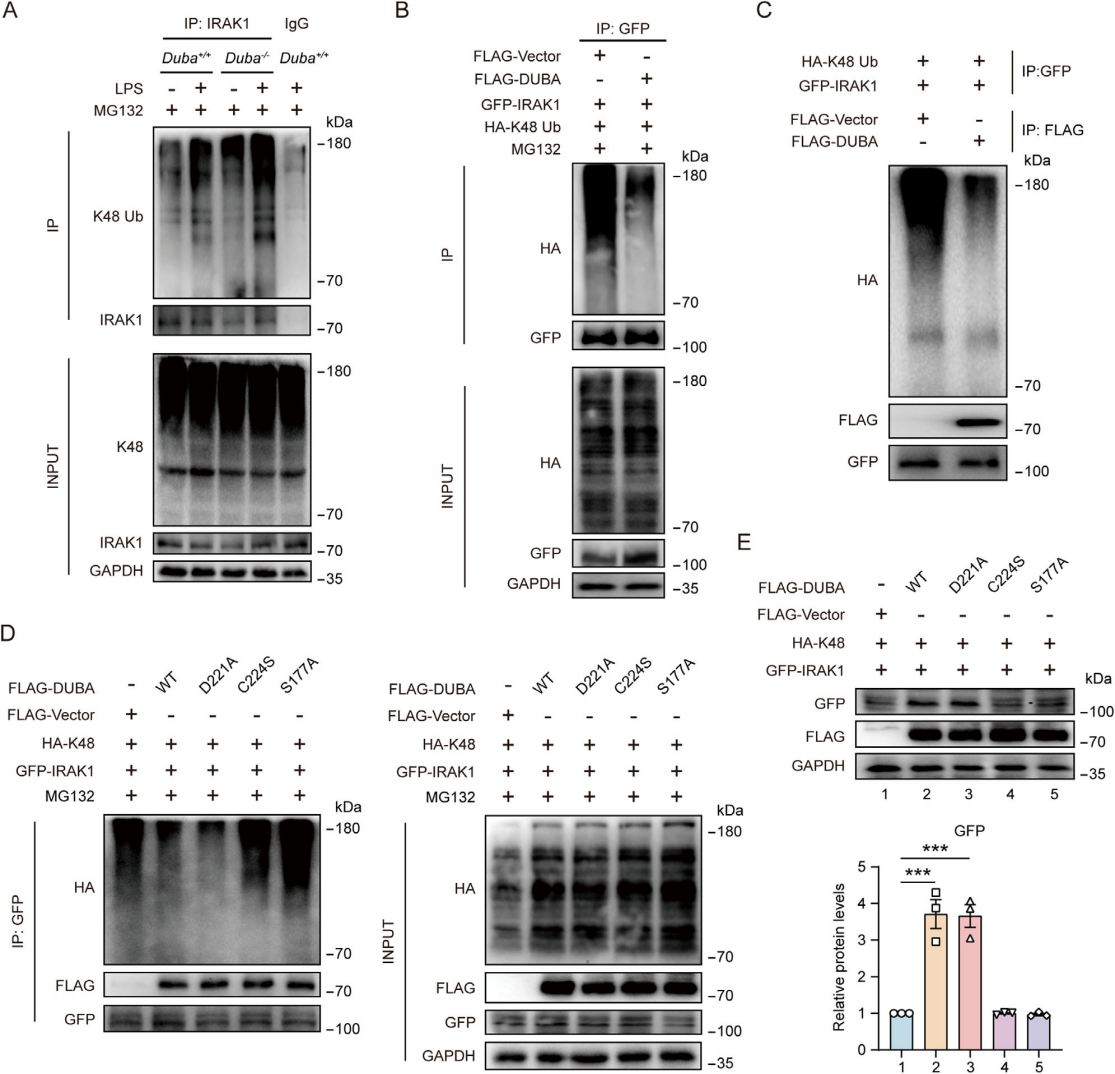
DUBA removes K48‐specific ubiquitin chains from IRAK1. A) *Duba* ^+/+^ and *Duba* ^‐/‐^ BV2 cells were treated with LPS (500 ng mL^−1^) for 30 min or left untreated, followed by MG132 (5 µM) treatment for 6 h. Whole‐cell lysates were immunoprecipitated with indicated antibodies and then analyzed by Western blot. B) BV2 cells were transfected with indicated plasmids for 24 h, followed by treatment with MG132 (5 µM) for 6 h. Whole‐cell lysates were immunoprecipitated with anti‐GFP antibodies and then analyzed by Western blot. C) Representative immunoblots of the in vitro deubiquitination assay. D) BV2 cells were transfected with indicated plasmids for 24 h, followed by treatment with MG132 (5 µM) for 6 h. Whole‐cell lysates were immunoprecipitated with anti‐GFP antibodies and then analyzed by Western blot. E) BV2 cells were transfected with indicated plasmids for 24 h. Whole‐cell lysates were analyzed by Western blot. Representative immunoblots (upper panel) and quantification (lower panel) are shown (n = 3). Mean ± SEM. ***p < 0.001.

Considering that IRAK1 is a K48‐ubiquitinated protein prior to degradation in the proteasome and that DUBA inhibited the proteasomal degradation of IRAK1, we next examined the effect of DUBA on IRAK1 ubiquitination. Deletion of DUBA markedly increased the K48‐specific polyubiquitination of IRAK1 (**Figure** **5**A). Consistently, overexpression of DUBA decreased the total and K48 ubiquitination of IRAK1 (Figure 5B; Figure S7A, Supporting Information). However, DUBA exerted no impact on the K11‐ and K63‐linked polyubiquitination of IRAK1 (Figure S7B,C, Supporting Information). To determine whether DUBA can cleave K48‐specific polyubiquitin chains linked to IRAK1, we performed an in vitro deubiquitination assay and found that DUBA was able to directly remove K48‐linked polyubiquitin chains from the ubiquitinated IRAK1 (Figure 5C). Furthermore, we found that the D221A mutant of DUBA could deubiquitinate IRAK1 as efficiently as wildtype (WT) DUBA whereas the C224S and S177A mutants lost the ability to deubiquitinate IRAK1 (Figure 5D), indicating that the deubiquitinating activity of DUBA toward IRAK1 is mediated by the C224 and S177 active sites. Since the C224S and S177A mutants could not K48 deubiquitinate IRAK1, they also failed to stabilize IRAK1 when overexpressed (Figure 5E).

### 2.6 Deficiency of Microglial DUBA Alleviates Depression‐Like Behavior in LPS‐Treated Mice

**Figure 6:**
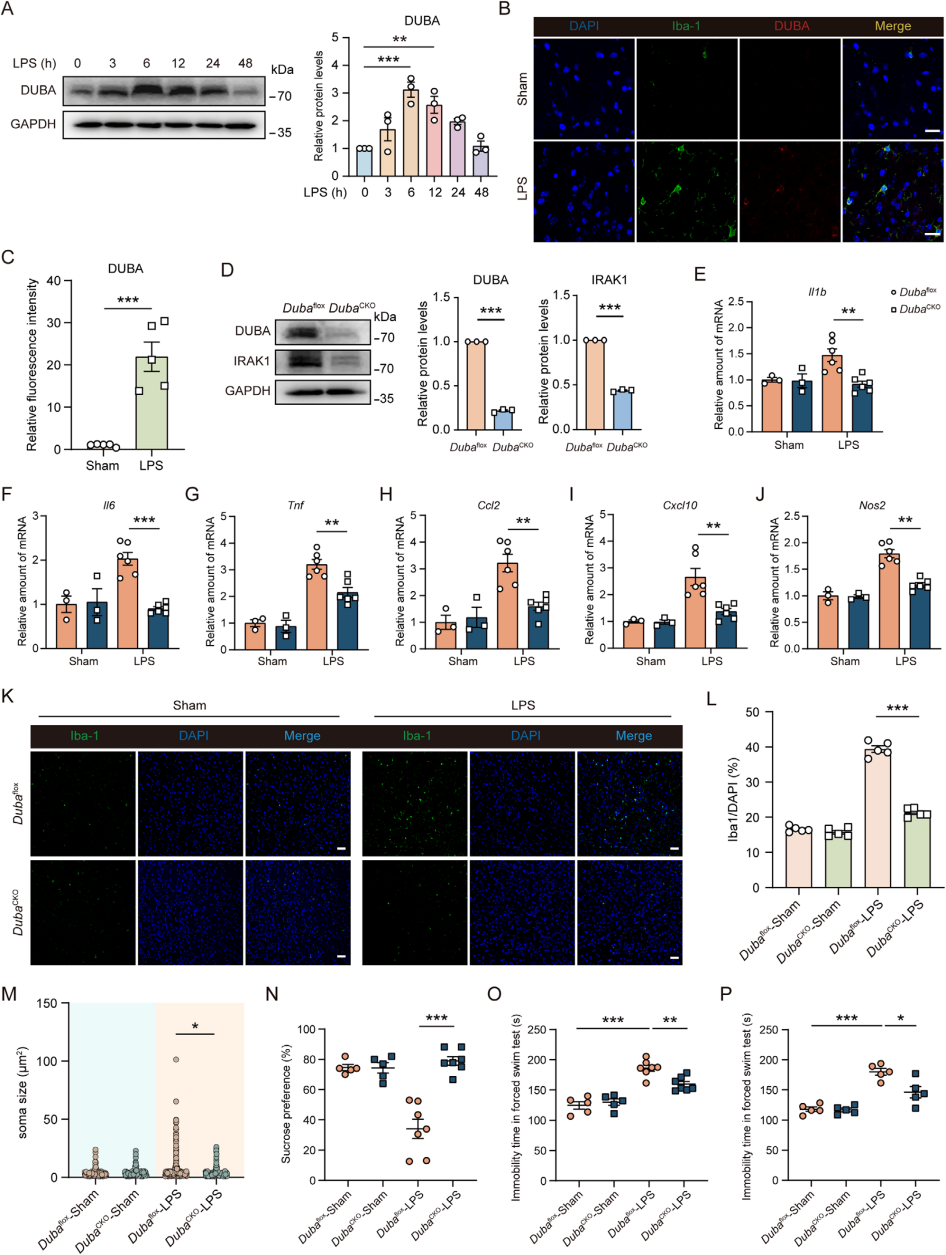
DUBA deficiency in microglia alleviates LPS‐induced depression in mice. A) Primary microglia were stimulated with LPS (500 ng mL^−1^) for indicated periods of time, followed by Western blot analysis. Representative immunoblots (left panel) and quantification (right panel) are shown (n = 3). Mean ± SEM. **p < 0.01, ***p < 0.001. B,C) Representative immunofluorescence staining (B) of DUBA (red), Iba‐1 (green), and DAPI (blue) in the brain of mice intraperitoneally injected with LPS (0.5 mg kg^−1^) or PBS (Sham) for 7 consecutive days. The quantification of relative fluorescence intensity of DUBA (C) is shown (n = 5). Scale bar, 20 µm. Mean ± SEM. ***p < 0.001. D) Western blot was performed to determine DUBA and IRAK1 protein levels in primary microglia from *Duba* ^flox^ and *Duba* ^cko^ mice. Representative immunoblots (left panel) and quantification (right) are shown (n = 3). Mean ± SEM. ***P < 0.001. E‐J) *Duba* ^flox^ and *Duba* ^cko^ mice were intraperitoneally injected with LPS (0.5 mg kg^−1^) daily for 7 consecutive days. Transcription of *Il1b* (E), *Il6* (F), *Tnf* (G), *Ccl2* (H), *Cxcl10* (I), and *Nos2* (J) in the brain was analyzed by qRT‐PCR (n = 3‐6). Mean ± SEM. **P < 0.01, ***P < 0.001. K‐M) Representative immunofluorescence staining (K) as well as percentages (L) and soma size (M) of Iba‐1^+^ cells in the brain of *Duba* ^flox^ and *Duba* ^cko^ mice intraperitoneally injected with LPS (0.5 mg kg^−1^) daily for 7 consecutive days. Scale bar, 50 µm. *P < 0.05, ***p < 0.001. N,O) *Duba* ^flox^ and *Duba* ^cko^ mice were intraperitoneally injected with LPS (0.5 mg kg^−1^) daily for 7 consecutive days. Thereafter, the sucrose preference test (N) and forced swim test (O) were performed (n = 5‐7). Mean ± SEM. **P < 0.01, ***P < 0.001. P) *Duba* ^flox^ and *Duba* ^cko^ mice were intraperitoneally injected with LPS (1 mg kg^−1^). Eight hours later, mice were assessed by the forced swim test and immobility time was recorded (n = 5). Mean ± SEM. *P < 0.05, ***P < 0.001.

Consistent with the results obtained with BV2 cells (Figure 1A–D), LPS stimulation rapidly upregulated DUBA protein levels in primary microglia in vitro (**Figure** **6**A). To validate whether DUBA can be upregulated in microglia upon LPS stimulation in vivo, we treated C57BL/6 mice with low‐dose LPS for 7 days and then analyzed DUBA expression in microglia. In agreement with in vitro findings (Figures 1A ‐D and 6A), DUBA was also markedly upregulated in microglia in LPS‐treated mice (Figure 6B,C). To study the in vivo function of microglial DUBA, we generated inducible microglia‐specific DUBA knockout (*Duba* ^cko^) mice by crossing *Duba* ^flox^ mice with Cx3cr1‐Cre^ERT2^ mice (Figure S8A, Supporting Information). After tamoxifen treatment, DUBA was efficiently ablated in microglia of *Duba* ^cko^ mice (Figure S8B,C, Supporting Information). Moreover, DUBA deficiency was associated with reduced IRAK1 expression in primary microglia (Figure 6D). Next, we analyzed the effect of microglial DUBA on LPS‐induced neuroinflammation. After LPS treatment, significantly lower levels of proinflammatory mediators were detected in the brain of *Duba* ^cko^ mice (Figure 6E–J). Consistently, both the number and activation of microglia were conspicuously reduced in LPS‐stimulated *Duba* ^cko^ mice as compared to *Duba* ^flox^ mice (Figure 6K‐M; Figure S9, Supporting Information), showing that the specific deletion of DUBA in microglia reduced neuroinflammation in LPS‐treated mice. Considering that LPS‐induced neuroinflammation leads to depression‐like behavior in mice, we then assessed the impact of microglial DUBA on behavioral changes in LPS‐treated mice. The sucrose preference test and forced swim test showed that deficiency of microglial DUBA significantly alleviated depression‐like behavior in LPS‐treated mice (Figure 6N–P). Consistent with previous in vitro findings, these in vivo results reveal that DUBA enhances LPS‐induced microglia‐mediated neuroinflammation in mice, implying that microglia‐derived DUBA may also play a role in other neuroinflammation‐associated diseases.

### 2.7 Ischemic Stroke Upregulates Microglial DUBA Expression and Deletion of Microglial DUBA Attenuates Ischemic Injury In Mice

**Figure 7:**
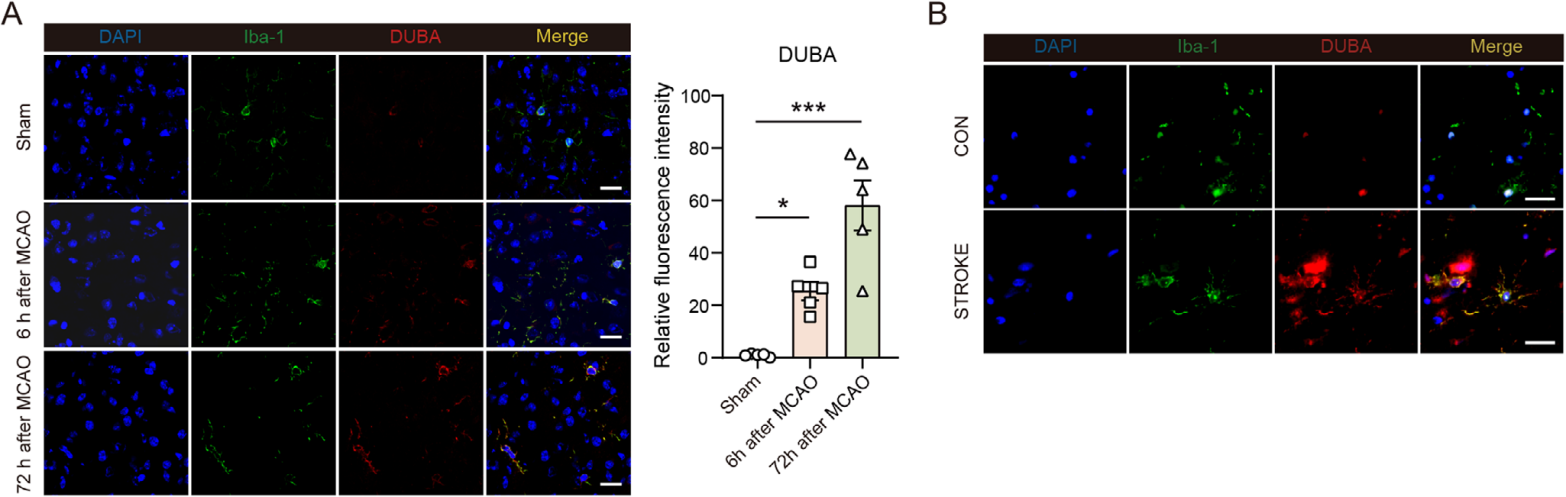
Upregulation of microglial DUBA in ischemic stroke and depression. A) Representative immunofluorescence staining of DUBA (red), Iba‐1 (green), and DAPI (blue) in the ischemic penumbra at indicated time points after MCAO (left panel). The right panel shows the quantification of relative fluorescence intensity of DUBA (n = 5). Scale bar, 20 µm. Mean ± SEM. *P < 0.05, ***p < 0.001. B) Representative immunofluorescence staining of DUBA (red), Iba‐1 (green), and DAPI (blue) in a 61‐year old female human control (CON) brain and in the ischemic penumbra of a 67‐year old female patient with an 1‐day old infarct (STROKE). Scale bar, 40 µm.

**Figure 8:**
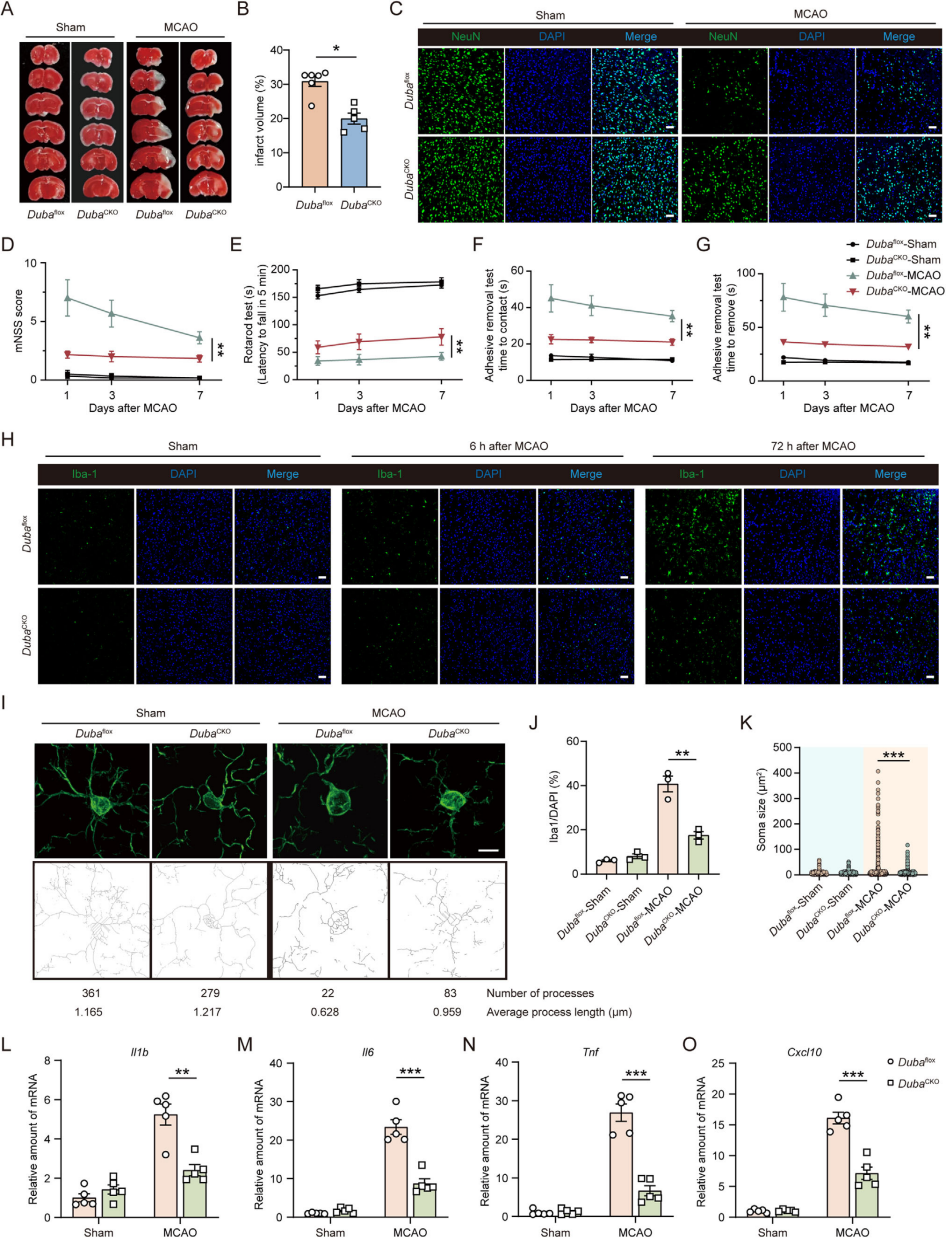
DUBA deficiency in microglia alleviates ischemic stroke injury in mice. A,B) Representative TTC staining (A) and quantification of infarct volume (B) on day 7 after operation (n = 5‐6). Mean ± SEM. *P < 0.05. C) Representative NeuN immunofluorescence staining of the ischemic penumbra on day 3 after operation. Scale bar, 50 µm. D‐G) Neurobehavioral functions of *Duba* ^flox^ and *Duba* ^cko^ mice were evaluated by the mNSS test (D), rotarod test (E), and adhesive removal test (F‐G) on days 1, 3, and 7 after operation (n = 6). Mean ± SEM. **P < 0.01. H) Representative Iba‐1 immunofluorescence staining of the ischemic penumbra. Scale bar, 50 µm. I) Representative Z‐stack images of Iba1^+^ microglia in the ischemic penumbra at 6 h after operation. Scale bar, 5 µm. J,K) Percentages (J) and soma size (K) of Iba‐1^+^ cells in the ischemic penumbra at 72 h after operation (n = 3). Mean ± SEM. **P < 0.01, ***P < 0.001. L‐O) Transcription levels of *Il1b* (L), *Il6* (M), *Tnf* (N), and *Cxcl10* (O) in the ischemic cerebral hemisphere were determined by qRT‐PCR at 6 h after operation (n = 5).

**Figure 9:**
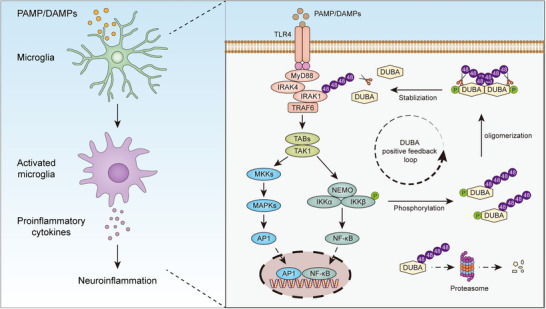
Schematic illustration of the role of microglial DUBA in neuroinflammation. In response to DAMPs or PAMPs, activated microglia produce proinflammatory cytokines to establish neuroinflammation. Activation of microglia by PAMP/DAMPs induces the stabilization and accumulation of DUBA, which subsequently acts as a positive feedback modulator to sustain proinflammatory signal transduction in microglia.

Ischemic stroke is a common yet severe disease and neuroinflammation is a key factor contributing to ischemic stroke injury.^[^ ^18^ ^]^ After an ischemic stroke, microglia recognize DAMPs leaked from necroptotic neurons through TLRs, resulting in the production of neuroinflammatory mediators.^[^ ^18^, ^19^ ^]^ We found that, similar to LPS treatment, an ischemic insult in mice induced the upregulation of microglial DUBA in the penumbra (**Figure** **7**A). In contrast, DUBA was not upregulated in neurons, astrocytes, or oligodendrocytes after ischemic stroke (Figure S10A‐C, Supporting Information). Importantly, DUBA expression was also remarkably upregulated in microglia in the ischemic penumbra of patients with ischemic stroke (Figure 7B), implying a potential role of DUBA in ischemic stroke injury. Therefore, we induced cerebral ischemia in *Duba* ^flox^ and *Duba* ^cko^ mice through middle cerebral artery occlusion (MCAO). After MCAO, cerebral infarct size in *Duba* ^cko^ mice was significantly smaller than in *Duba* ^flox^ mice (**Figure** **8**A,B). Moreover, compared with *Duba* ^flox^ mice, more neurons were preserved in the ischemic penumbra of *Duba* ^cko^ mice (Figure 8C; Figure S11, Supporting Information). Behavioral tests, including modified neurological severity score (mNSS), rotarod test, and adhesive removal test, revealed that MCAO‐induced neurological deficits were significantly ameliorated in *Duba* ^cko^ mice (Figure 8D–G). These results show that the specific deletion of DUBA in microglia attenuates ischemic stroke injury in mice. At 6 hours after MCAO, *Duba* ^cko^ and *Duba* ^flox^ mice had comparable numbers of neurons, astrocytes, and microglia in the ischemic penumbra (Figure 8H; Figure S12A,B, Supporting Information). However, at this early stage after MCAO, microglia in *Duba* ^cko^ mice had smaller soma size and more processes than that in *Duba* ^flox^ mice (Figure 8I; Figure S13A,B, Supporting Information), indicating that DUBA deficiency decreases MCAO‐induced microglial activation. In addition, at 72 hours after MCAO, both the number and activation of microglia were significantly reduced in the ischemic penumbra of *Duba* ^cko^ mice compared with *Duba* ^flox^ mice (Figure 8H,J,K; Figure S14, Supporting Information). Moreover, astrocyte activation was comparable between *Duba* ^cko^ and *Duba* ^flox^ mice at this time point (Figure S12C,D, Supporting Information). Activated microglia are a major source of neuroinflammatory cytokines and chemokines in the ischemic brain, and we found that DUBA deficiency impaired the ability of microglia to produce TNF‐α in the ischemic penumbra (Figure S15, Supporting Information). As a result, after MCAO, significantly lower levels of *Il1b*, *Il6*, *Tnf*, and *Cxcl10* were expressed in the brain of *Duba* ^cko^ mice compared with *Duba* ^flox^ mice (Figure 8L–O). Taken together, these findings show that DUBA exacerbates ischemic stroke injury by enhancing microglia‐mediated neuroinflammatory responses (**Figure** **9**).

## 3 Discussion

Microglia are crucial sentinels of the CNS and they are activated early in neuroinflammatory disorders. In the present study, we identified DUBA as a critical regulator of microglial activation under neuroinflammatory conditions. DUBA was expressed at low levels in microglia but rapidly upregulated in response to DAMPs or PAMPs, thereby potentiating the synthesis of neuroinflammatory mediators to promote inflammatory responses. These unique features of DUBA make it an attractive candidate to be exploited as a druggable target for modulating the neuroinflammatory response.

Previous reports have shown that LPS induces the upregulation of DUBA but does not alter *Duba* transcription.^[^ ^12^, ^15^ ^]^ However, the underlying mechanism remains unknown. Here, we found that DUBA protein was constantly K48 ubiquitinated and degraded in the 26S proteasome, maintaining DUBA at low levels in resting cells. Upon PRR activation, DUBA was activated through phosphorylation and then self‐deubiquitinated by cleaving its K48‐linked polyubiquitin chains, leading to DUBA stabilization and accumulation. Therefore, our findings resolved the long‐standing question of why LPS induced DUBA upregulation without affecting its transcription.^[^ ^12^, ^15^ ^]^ The ubiquitination‐mediated regulation of DUBA stability is exempted from time‐consuming transcription and translation, allowing for a swift alteration in protein abundance in response to stimuli. This unique regulatory mechanism also highlights the importance of DUBA in PRR‐mediated inflammatory responses.

In line with a previous report,^[^14b^]^ we found that DUBA enhanced NF‐κB and MAPK signaling induced by LPS. Furthermore, we discovered that DUBA promoted LPS‐induced signaling by stabilizing IRAK1. Upon TLR activation, IRAK1 is recruited to the Myddosome and then interacts with TRAF6, resulting in TRAF6 activation through self‐K63‐ubiquitination.^[^ ^20^ ^]^ In human macrophages and murine microglia, LPS‐induced signaling and cytokine production are critically dependent on IRAK1.^[^ ^21^ ^]^ Notably, IRAK1 can be modified by both K48‐ and K63‐linked polyubiquitination.^[^17a^]^ K63‐linked IRAK1 polyubiquitination facilitates TLR‐induced signal transduction whereas K48 ubiquitination labels IRAK1 for degradation in the 26S proteasome.^[^17b^,^22^]^ In this study, we revealed that the K48 ubiquitination of IRAK1 was inhibited by DUBA.

DUBA requires phosphorylation at the S177 residue for its enzymatic activity.^[^ ^15^ ^]^ Consistently, we found that the S177 site of DUBA was indispensable for deubiquitinating itself and IRAK1. After phosphorylation, DUBA acquires the deubiquitinating activity against K48 and K63 linkages.^[^ ^23^ ^]^ The enzymatic activity of phosphorylated DUBA is exerted by D221 and C224 active residues, both of which were required for DUBA self‐deubiquitination. In comparison, only the C224 residue was needed for deubiquitinating IRAK1. In good agreement, previous studies have shown that the C224 site is essential for DUBA to deubiquitinate TRAF3, TAK1, and STING.^[^ ^12^, ^14^, ^24^ ^]^

Previously, A20, another DUB of the OTU subfamily, has been identified as one of the most potent inhibitors of TLR signaling.^[^ ^25^ ^]^ In addition, A20 serves as a critical gatekeeper of microglial activation under homeostatic conditions and A20 deficiency in microglia aggravates CNS immunopathology.^[^10a^,^10c^]^ Therefore, although A20 and DUBA share sequence and domain conservation, they exert opposite functions in TLR signaling and microglial activation. Unlike DUBA, A20 is upregulated after PRR activation through *de novo* gene transcription, which is a common mechanism for most proteins.^[^ ^26^ ^]^ In our study, DUBA was found to be the only DUB of the OTU family that could be rapidly upregulated upon LPS stimulation, further highlighting the particularity and importance of DUBA in TLR signaling.

Given that global deletion of DUBA is lethal in mice, cell‐specific DUBA knockout mice are used to study the in vivo function of DUBA.^[^ ^27^ ^]^ Previous studies have found that DUBA affects intestinal inflammation, innate antiviral and antitumor immunity, and kidney inflammation by specifically regulating T cells, macrophages, and podocytes, respectively.^[^ ^13^, ^14^, ^24^ ^]^ In humans, genetic variants of *Duba* are associated with primary biliary cholangitis,^[^ ^28^ ^]^ highlighting the significance of DUBA in inflammatory diseases. Moreover, considering that DUBA protein levels are rapidly upregulated in response to proinflammatory stimuli, DUBA may serve as a potential biomarker for inflammatory conditions. In the present study, we found that specific deletion of DUBA in microglia diminished DAMP‐ and PAMP‐induced neuroinflammation, thereby ameliorating LPS‐induced depression‐like behavior and ischemic stroke injury, expanding the spectrum of inflammatory diseases regulated by DUBA. In addition, chronic neuroinflammation mediated by the cGAS‐STING pathway contributes to aging‐related neurodegeneration and multiple neurodegenerative diseases, such as amyotrophic lateral sclerosis, Alzheimer's disease, Parkinson's disease, and Huntington's disease.^[^ ^29^ ^]^ Given that DUBA potentiates cGAS‐STING activation by stabilizing STING,^[^ ^24^ ^]^ it is also possible that DUBA promotes neurodegenerative diseases by regulating chronic neuroinflammation. Specific and potent small‐molecule inhibitors for DUBA, which have not yet been developed, may be beneficial for the treatment of neuroinflammation. In summary, this study provides essential insights into the regulatory mechanism of microglial activation under neuroinflammatory conditions and implies that inhibition of DUBA may be beneficial for treating neuroinflammatory diseases.

## 4 Experimental Section

### Mice

*Duba* ^flox^ and Cx3cr1‐Cre^ERT2^ mice on the C57BL/6 background were provided by GemPharmatech. In *Duba* ^flox^ mice, the exon 2 of *Duba* was flanked by loxp sites. *Duba* ^flox^ and Cx3cr1‐Cre^ERT2^ mice were crossed to generate Cx3cr1‐Cre^ERT2^ *Duba* ^flox^ (*Duba* ^cko^) mice. Genotyping was performed by PCR on tail DNA with the following primers (5' to 3'): JS00028‐Otud5‐5wt‐tF1A GTGACCACCTCACCAGTATAGGCT, JS00028‐Otud5‐5wt‐tR1A GCCAGAAGCAGACAGCTTGACCT (wt: 327 bp, flox: 405 bp); Cx3crlCreER‐Common (12266) AAGACTCACGTGGACCTGCT, Cx3crlCreER‐Mutant Reverse (14314) CGGTTATTCAACTTGCACCA, Cx3crlCreER‐WT Reverse (16221) AGGATGTTGACTTCCGAGTTG (ko: 300 bp, wt: 695 bp). All primers were synthesized by Sangon Biotech. Adult *Duba* ^flox^ and *Duba* ^cko^ mice were intraperitoneally injected with 100 mg kg^−1^ tamoxifen (CAT#HY‐13757A, MedChemExpress) daily for 7 consecutive days to induce gene recombination. Mice were kept in an SPF environment with standard food and water, and animal experiments were approved by the Animal Policy and Welfare Committee of Wenzhou Medical University (Approval number: wydw2024‐0297).

### Cell Culture and Treatment

BV2 cells were cultured in DMEM medium (CAT#11965118, Gibco) containing 1% penicillin/streptomycin (CAT# P1400, Solarbio) and 10% fetal bovine serum (FBS; CAT#FSD500, ExCell Bio). *Duba* ^‐/‐^ BV2 cells were generated using the CRISPR/Cas9 technology (DUBA‐gRNA: AGAGATGTACAACCGTCCTG) as previously described.^[^ ^30^ ^]^ Single‐cell *Duba* ^‐/‐^ BV2 clones were selected using Blasticidin S (CAT#60218ES10, Yeasen Biotechnology) and verified by Western blot. For the overexpression or knockdown of proteins, cells were transfected with indicated plasmids (Genechem) or DUBA siRNA (5'‐CCGGAATATCCACTATAAT‐3', RiboBio) using Lipofectamine 3000 (CAT#L3000015, Thermo Fisher Scientific). Cells were treated with LPS (CAT#P1010, Solarbio), MG132 (CAT#HY‐13259, MedChemExpress), chloroquine (CAT#HY‐17589A, MedChemExpress), cycloheximide (CAT#HY‐12320, MedChemExpress), actinomycin D (CAT#HY‐17559, MedChemExpress), IKKβ inhibitor (CAT#HY‐13802, MedChemExpress), IKK inhibitor (CAT#HY‐13453, MedChemExpress), p38 inhibitor (CAT#HY‐10578, MedChemExpress), ERK inhibitor (CAT#HY‐12028, MedChemExpress), and/or JNK inhibitor (CAT#HY‐13319, MedChemExpress) as described in figure legends.

### MCAO Model

MCAO was performed as previously described.^[^11b^]^ After anesthetization of mice with 2% isoflurane, the neck skin was incised longitudinally to expose the left common carotid artery (CCA), external carotid artery (ECA), and internal carotid artery (ICA). A MCAO suture (CAT#MSMC19B110PK50, RWD life science) was inserted into the left ICA through an incision in the left ECA, blocking blood flow to the middle cerebral artery (MCA) with the silicone‐coated tip of the suture. Local cerebral blood flow was monitored using a laser Doppler flowmeter, and the cerebral blood flow dropped to below 25% of baseline when the MCA was properly obstructed. One hour later, the suture was removed for reperfusion, restoring cerebral blood flow to over 70% of baseline. Except for suture insertion, mice in the sham group underwent the same surgical procedure as those in the MCAO group.

### 2,3,5‐Triphenyltetrazolium Chloride (TTC) Staining

Brains were extracted from euthanized mice and then sliced into 1 mm‐thick sections in a mold (CAT#68707, RWD life science). Thereafter, the sections were stained in 2% TTC solution (CAT#298‐96‐4, Sigma‐Aldrich) for 10 min in the dark at 37 °C, followed by fixation in 4% paraformaldehyde (CAT#P1110) for 5 min. Images were taken with a digital camera and analyzed using ImageJ to calculate the infarct area.

### Behavioral Assessment

Neurological deficits induced by MCAO were assessed by mNSS, rotarod test, and adhesive removal test on days 1, 3, and 7 after the operation. All mice were trained for 3 consecutive days before the operation. For the induction of depression‐like behavior, mice were intraperitoneally injected with LPS (CAT#297‐473‐0, Sigma–Aldrich) at the concentration of 0.75 mg kg^−1^ once or 0.5 mg kg^−1^ once per day for 7 consecutive days. Depression was evaluated by sucrose preference test and forced swim test. Mice in the sham group were intraperitoneally injected with an equal volume of PBS. An investigator who was blinded to mouse identity carried out the behavioral assessment.

### Human Tissue

*Postmortem* paraffin‐embedded brain tissue specimens from two stroke cases and one non‐stroke case were obtained from the Department of Pathology, Odense University Hospital. Parallel tissue sections were used in previous studies.^[^11b^,^31^]^ The use of human material was approved by the Regional Ethical Committee in the Region of Southern Denmark (S‐20220018) and was performed in agreement with the declaration of Helsinki.

### Western Blot

Protein was extracted from cultured cells or animal tissue using cell protein extraction reagent (CAT#AR0103, BOSTER Biological Technology) and mammalian tissue protein extraction reagent (CAT#AR0101, BOSTER), respectively. Protein samples were quantified with Quick Start Bradford 1× Dye Reagent (CAT#5000205, BIO‐RAD) and then denatured at 100 °C for 10 min in 5× loading buffer (FD006, Hangzhou Fude Biological Technology). The protein samples were separated by sodium dodecyl sulfate‐polyacrylamide gel electrophoresis and subsequently transferred to polyvinylidene fluoride membranes (CAT#10600023, Cytiva). Thereafter, the membranes were incubated in 5% skim milk (CAT#1172GR500, BioFroxx) at room temperature for 90 min, followed by overnight incubation with primary antibodies at 4 °C. After that, corresponding horse radish peroxidase‐conjugated secondary antibodies were added and incubated at room temperature for 1 h. The immunoblots were developed using NcmECL Ultra (CAT#P10300, New Cell & Molecular Biotech), and the Fusion FX.EDGE system (Vilber) was applied to capture images. All antibodies used in this study are described in Supplementary Table S1.

### BS3 Cross‐Linking

After LPS stimulation, cells were lysed for protein isolation. Cell lysates were incubated with 3 mM bis (sulfosuccinimidyl) suberate (BS3; CAT#82436‐77‐9, Sigma–Aldrich) at room temperature for 30 min. Subsequently, 50 mM Tris buffer was added and incubated at room temperature for 15 min to terminate the cross‐linking reaction. The protein samples were then denatured and analyzed using Western blot.

### Co‐Immunoprecipitation

Protein samples were prepared as described in “Western blot”. Non‐specific binding proteins were eliminated from the protein samples by incubation with BeyoMag Protein A+G Magnetic Beads (CAT#P2108, Beyotime) at 4°C under gentle rotation for 2 h. After removing the beads by centrifugation, the samples were incubated with corresponding antibodies at 4 °C under gentle rotation overnight. The next day, magnetic beads were added to the samples and incubated at 4 °C under gentle rotation for 2 h. After incubation, the magnetic beads were washed five times with cold PBS and then collected for further analysis.

### Immunofluorescence Staining

Mouse brains were fixed in 4% paraformaldehyde for 48 h. Subsequently, the samples were embedded in Epredia Neg‐50 Frozen Section Medium (CAT#22‐110‐617, Thermo Fisher Scientific) and then cut into 5 or 20 µm slices. Cells cultured in glass bottom dishes (CAT#D29‐14‐0‐N, Cellvis) were fixed with 4% paraformaldehyde for 15 min and permeabilized with 0.5% Triton X‐100 (CAT#T8200, Solarbio). The samples were blocked with 5% BSA (CAT#SW3015, Solarbio) at 37 °C for 30 min, followed by incubation with primary antibodies at 4 °C overnight. The next day, the samples were incubated with corresponding secondary antibodies for 1 h in the dark at 37 °C. Thereafter, antifading mounting medium with DAPI (CAT#S2110, Solarbio) was added. Images were taken on a confocal microscope (CAT#FV3000, Olympus) and analyzed with ImageJ.

Human brain tissue was cut into 2 µm thick microtome sections, which were subsequently dewaxed in xylene and rehydrated in ethanol. Heat‐induced epitope retrieval was performed using citrate buffer (10 mM citrate, pH 6). Next, sections were bleached in Autofluorescence Eliminator Reagent (CAT#2160, Millipore) according to the manufacturer's guidelines. Sections were then rinsed and incubated with 10% FBS in Tris‐buffered saline (TBS) for 30 min before being incubated overnight with primary antibodies against DUBA and Iba‐1 diluted in TBS + 0.5% triton (TBS‐T). The next day, sections were rinsed in TBS‐T before being incubated with secondary antibodies. Finally, sections were rinsed in TBS before being mounted in ProLong Gold Antifade Mountant with DAPI (CAT#P36931, Thermo Fisher Scientific). Control sections for OTUD5 using rabbit IgG were devoid of signal. Images were captured using an Olympus BX53 microscope.

### In Vitro Deubiquitination Assay

BV2 cells were transfected with indicated plasmids for 24 h. DUBA and ubiquitinated target proteins were separately harvested from cell lysates by immunoprecipitation. The immunoprecipitated samples were washed sequentially with PBS and deubiquitination buffer (50 mM Tris‐HCl, 5% glycerol, 2 mM DTT, 2 mM ATP, and 5 mM MgCl_2_). Subsequently, immunoprecipitated proteins were mixed and then incubated in the deubiquitination buffer at 37°C for 2 h. After the reaction, the samples were harvested for Western blot analysis.

### qRT‐PCR

Total RNA from brain tissue or BV2 cells was isolated using RNAiso plus (CAT#9109, Takara). Subsequently, cDNA was generated by reverse transcription using PrimeScript RT reagent Kit with gDNA Eraser (RR047A, Takara). Quantitative real‐time PCR was performed with TB Green^®^ Premix Ex Taq (Tli RNaseH Plus) (CAT#RR420A, Takara) and corresponding primers on a QuantStudio 5 Real‐Time PCR System (Thermo Fisher Scientific). The primer sequences are described in Supplementary Table S2.

### Statistical Analysis

GraphPad Prism 8 (GraphPad) was used to perform statistics. Results were displayed as mean ± standard error of the mean (SEM), and the exact group size is shown in figures and/or legends. Quantitative values were calculated using two‐tailed unpaired Student's *t*‐test, one‐way Analysis of Variance (ANOVA), or two‐way ANOVA. Values of *p* < 0.05 indicate significance (* *p* < 0.05, ** *p* < 0.01, *** *p* < 0.001).

## Conflict of Interest

The authors declare no conflict of interest.

## Supporting information



Supporting Information

## Data Availability

The data that support the findings of this study are available from the corresponding author upon reasonable request.
