# Supplementary material for: Regulation of Neuroinflammation by Microglial DUBA‐IRAK1‐IKKβ Signaling Loop
Source: Adv Sci (Weinh). 2025 Aug 4;12(40):e03972. doi: 10.1002/advs.202503972 (PMC12561430; doi:10.1002/advs.202503972)
Supplement: Supplementary file 1 — Supporting Information [file ADVS-12-e03972-s001.docx]

**Supplementary materials**

**Fig. S1**


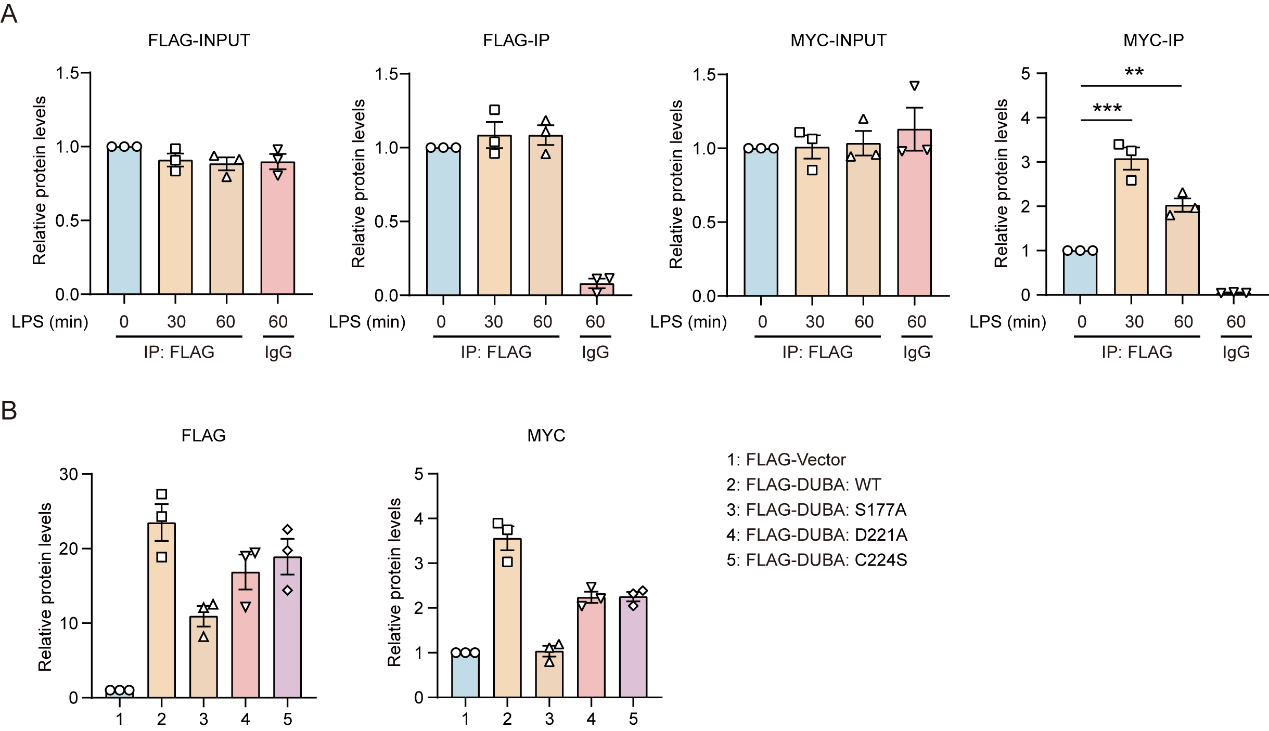


**Supplementary Figure 1. Quantification results for Figures 2C and 2G.**

(A-B) Quantification results for Figure 2C (A) and Figure 2G (B) (n = 3). Mean ± SEM. ** *P* < 0.01, *** *P* < 0.001.

**Fig. S2**


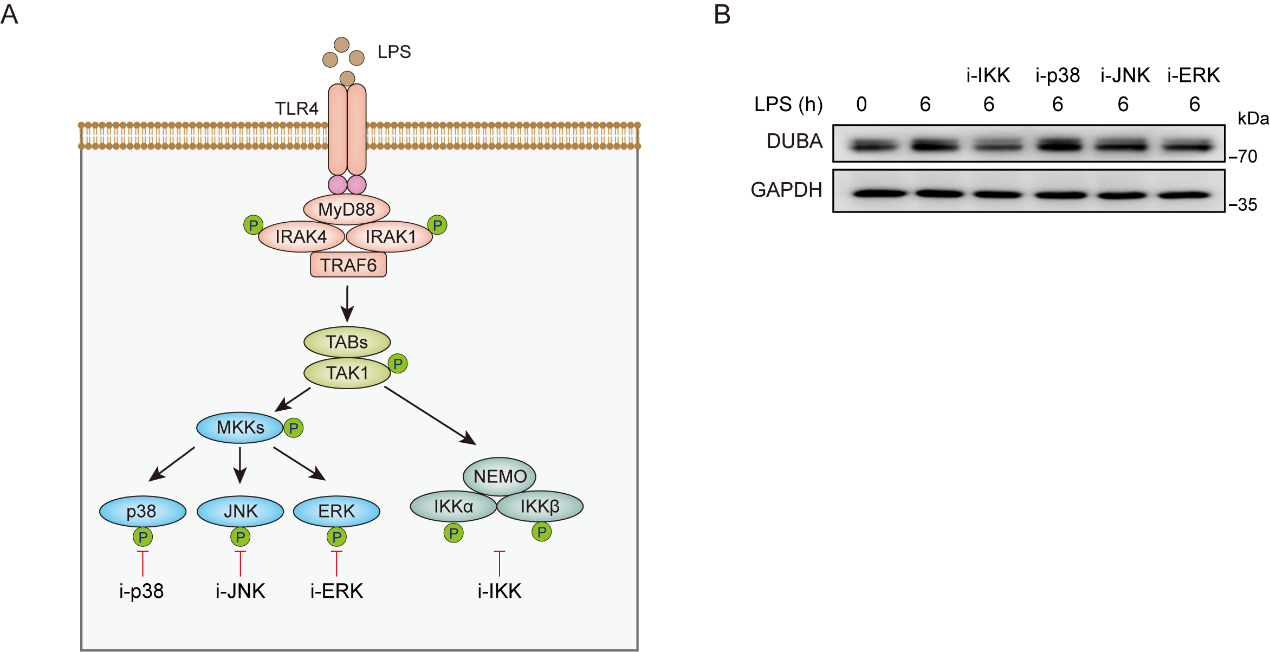


**Supplementary Figure 2. LPS induces the upregulation of DUBA through IKKs.**

(A) Schematic illustration of key kinases in the TLR4 signaling pathway. (B) BV2 cells were left untreated or stimulated with LPS (500 ng/ml), LPS + IKK inhibitor (1 μM), LPS + p38 inhibitor (1 μM), LPS + ERK inhibitor (1 μM), and LPS + JNK inhibitor (1 μM) for 6 h. Whole-cell lysates were analyzed by Western blot.

**Fig. S3**


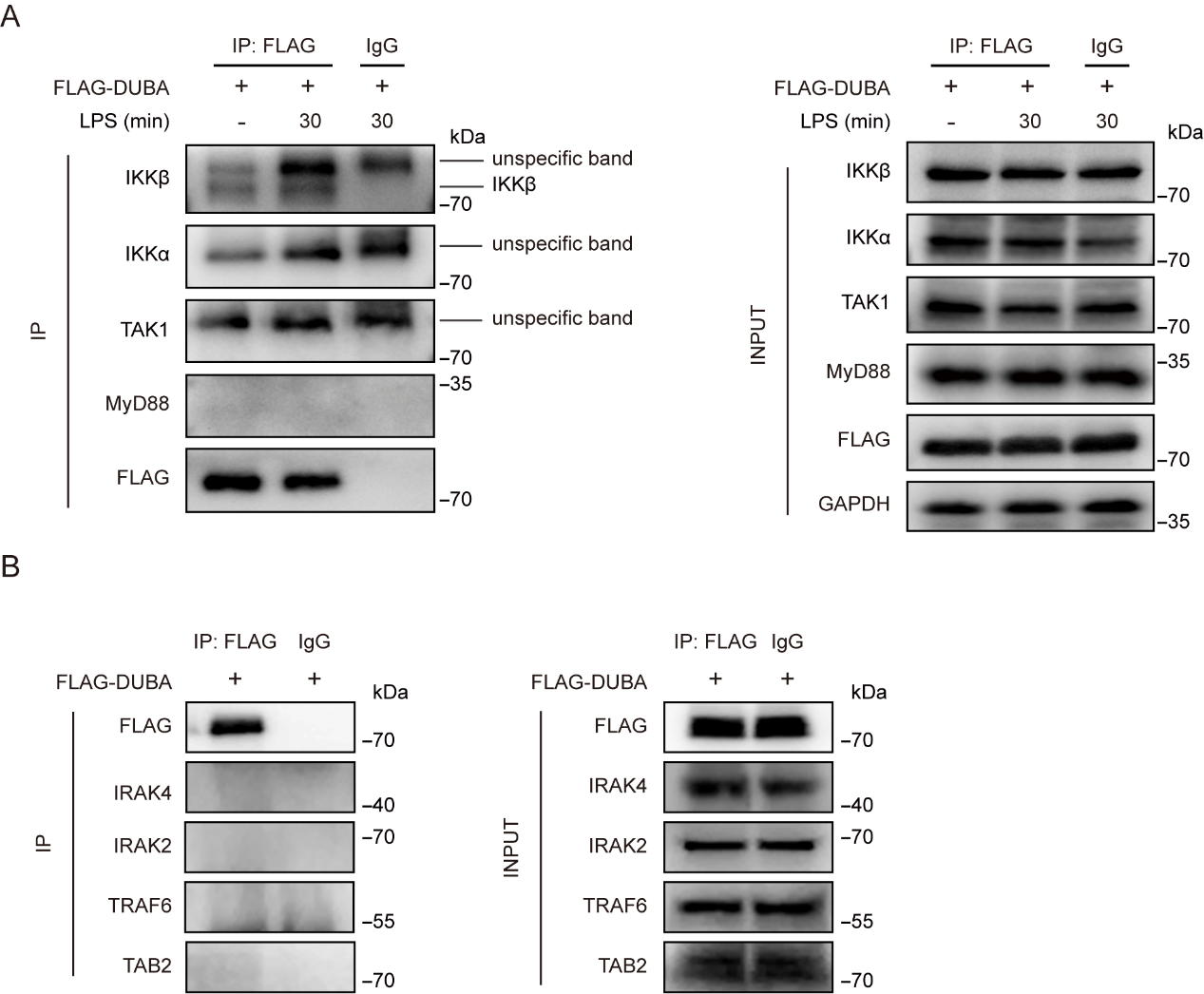


**Supplementary Figure 3. DUBA interacts with IKKβ.**

(A) BV2 cells were transfected with FLAG-DUBA plasmids for 24 h and then left untreated or stimulated with LPS (500 ng/mL) for 30 min. Proteins were immunoprecipitated from whole-cell lysates with indicated antibodies and then analyzed by Western blot. (B) BV2 cells were transfected with FLAG-DUBA plasmids for 24 h. After that, proteins were immunoprecipitated from whole-cell lysates with indicated antibodies and then analyzed by Western blot.

**Fig. S4**


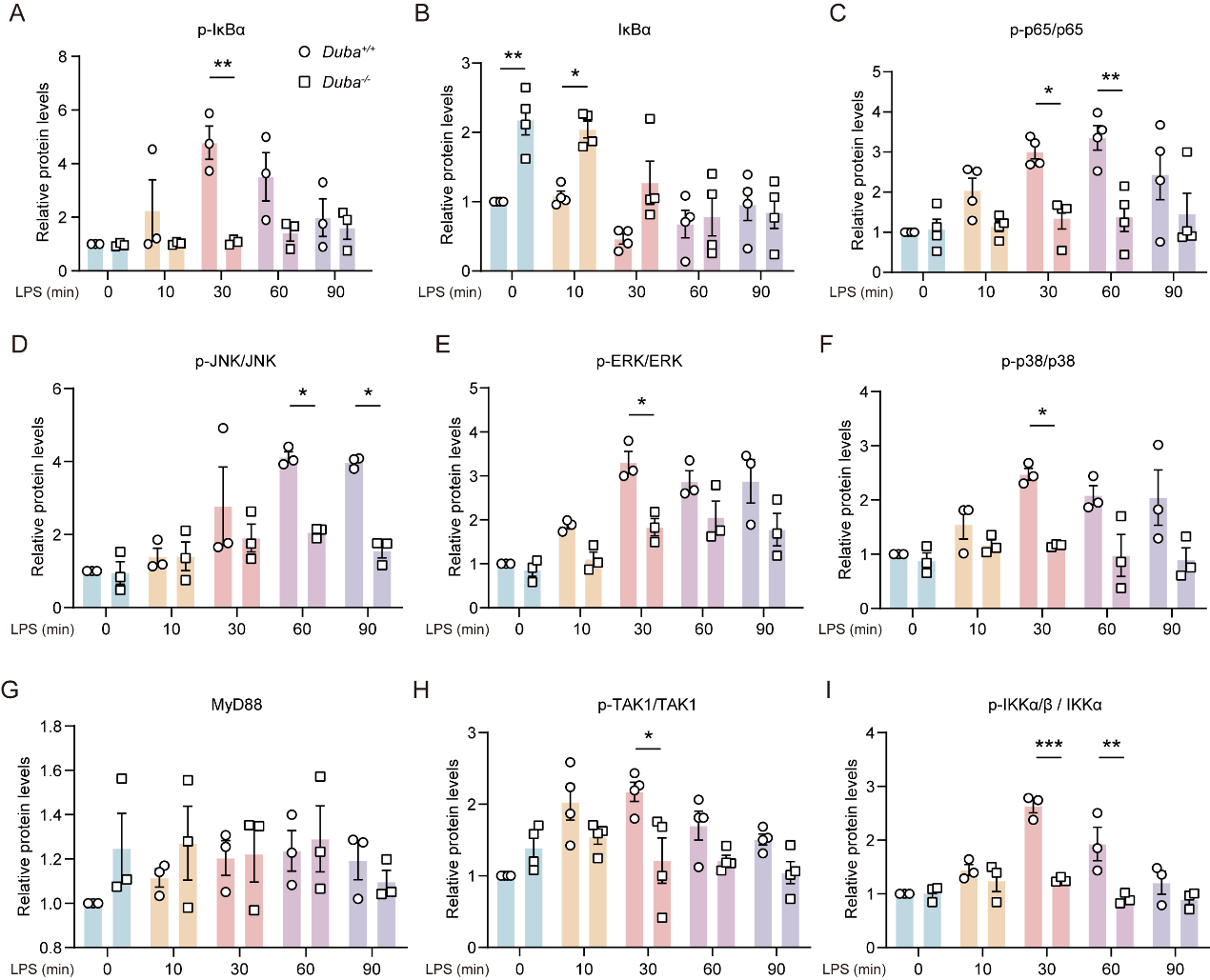


**Supplementary Figure 4. Quantification results for Figures 3G and 3I.**

(A-I) Quantification results for Figure 3G (A-F) and Figure 3I (G-I) (n = 3-4). Mean ± SEM. * *P* < 0.05, ** *P* < 0.01, *** *P* < 0.001.

**Fig. S5**


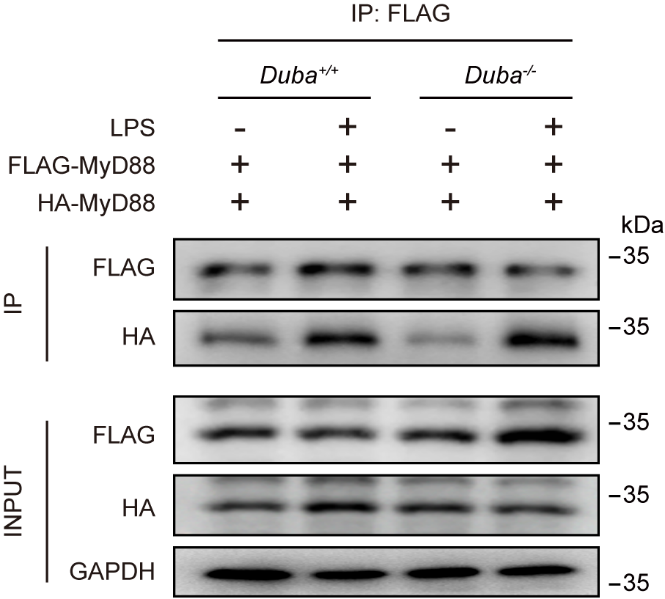


**Supplementary Figure 5. DUBA deficiency does not affect MyD88 oligomerization.**

BV2 cells were transfected with indicated plasmids for 24 h, followed by stimulation with LPS (500 ng/mL) for 0, 30 min. Proteins were immunoprecipitated from whole-cell lysates using anti-FLAG antibodies and analyzed by Western blot.

**Fig. S6**


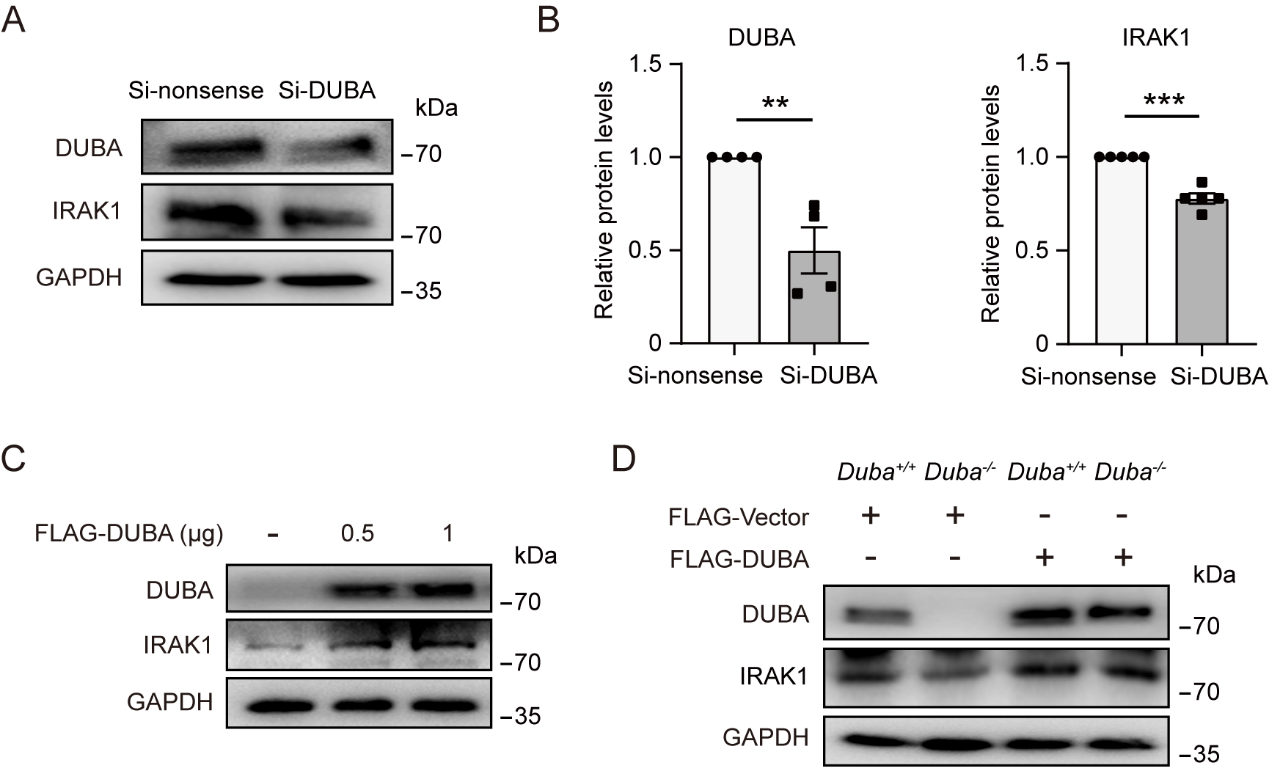


**Supplementary Figure 6. Protein levels of IRAK1 and DUBA are positively correlated.**

(A-B) BV2 cells were transfected with nonsense or DUBA siRNA for 72 h. Whole-cell lysates were analyzed by Western blot with indicated antibodies. Representative immunoblots (A) and quantification (B) are shown (n = 4-5). Mean ± SEM. ** *p* < 0.01. (C) BV2 cells were transfected with indicated amounts of FLAG-DUBA plasmids for 24 h before lysis. Whole-cell lysates were analyzed by Western blot. (D) *Duba*^+/+^ and *Duba*^-/-^ BV2 cells were transfected with indicated plasmids for 24 h. Cells were lysed and then analyzed by Western blot.

**Fig. S7**

**
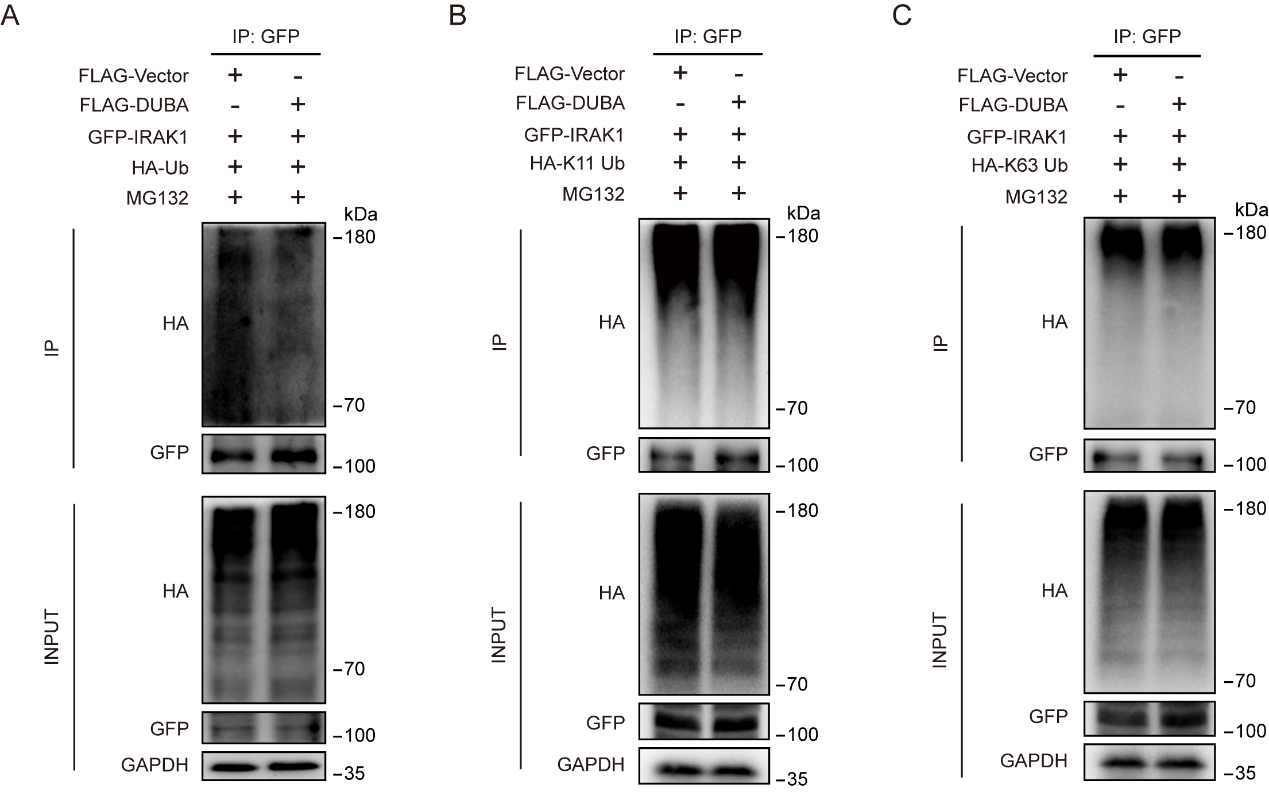
**

**Supplementary Figure 7. DUBA does not affect K11- and K63-linked polyubiquitination on IRAK1.**

(A-C) BV2 cells were transfected with indicated plasmids for 24 h, followed by treatment with MG132 (5 μM) for 6 h. Proteins were immunoprecipitated with anti-GFP antibodies and then analyzed by Western blot.

**Fig. S8**

**
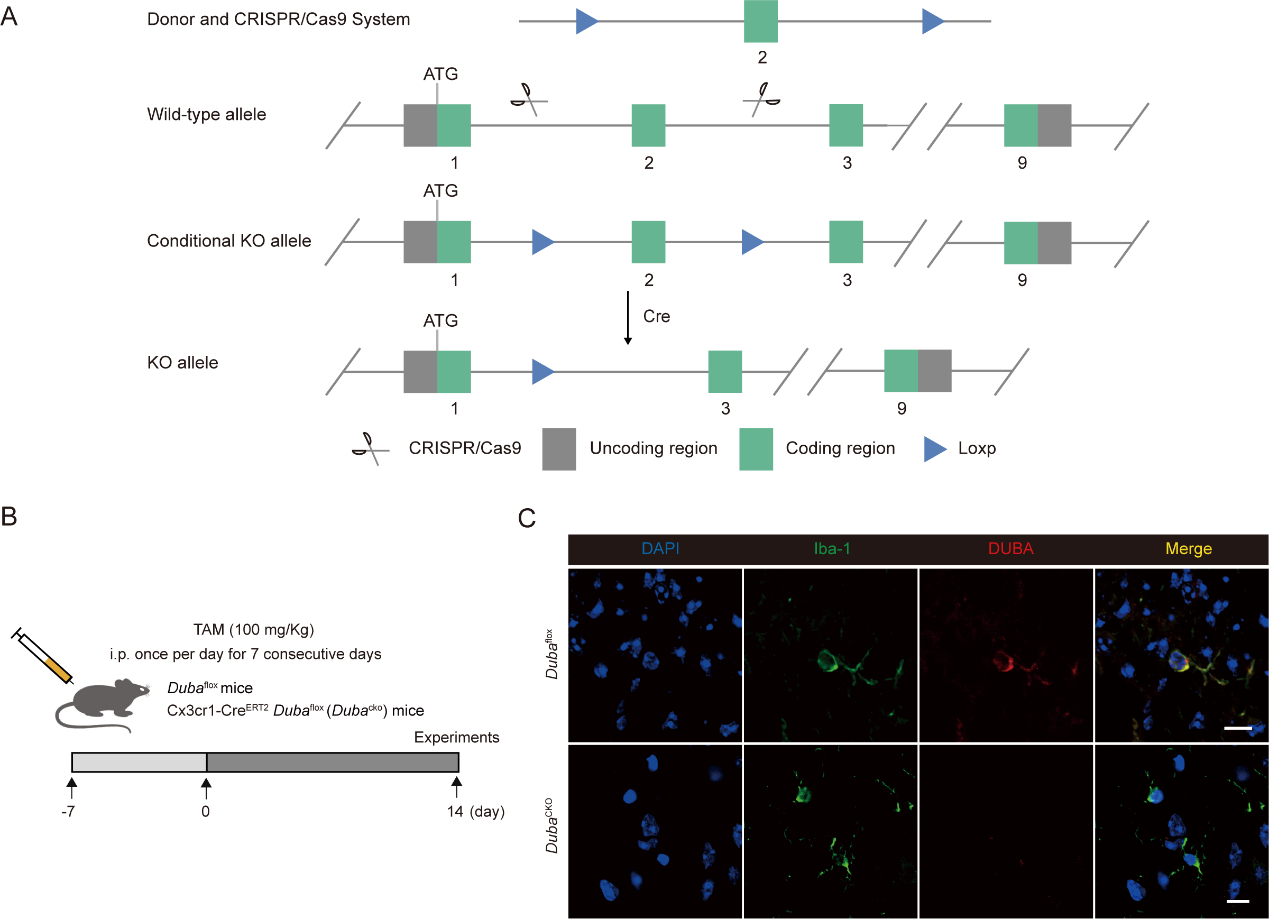
**

**Supplementary Figure 8. Generation and characterization of *Duba*^flox^ and *Duba*^cko^ mice.**

(A) Construction strategy for *Duba*^flox^ and Cx3cr1-Cre^ERT2^ *Duba*^flox^ (*Duba*^cko^) mice. (B) Schematic diagram showing the induction of DUBA deletion with tamoxifen. (C) Fourteen days after tamoxifen treatment, DUBA expression in microglia of *Duba*^flox^ and *Duba*^cko^ mice was confirmed by immunofluorescence. Scale bar, 10 μm.

**Fig. S9**


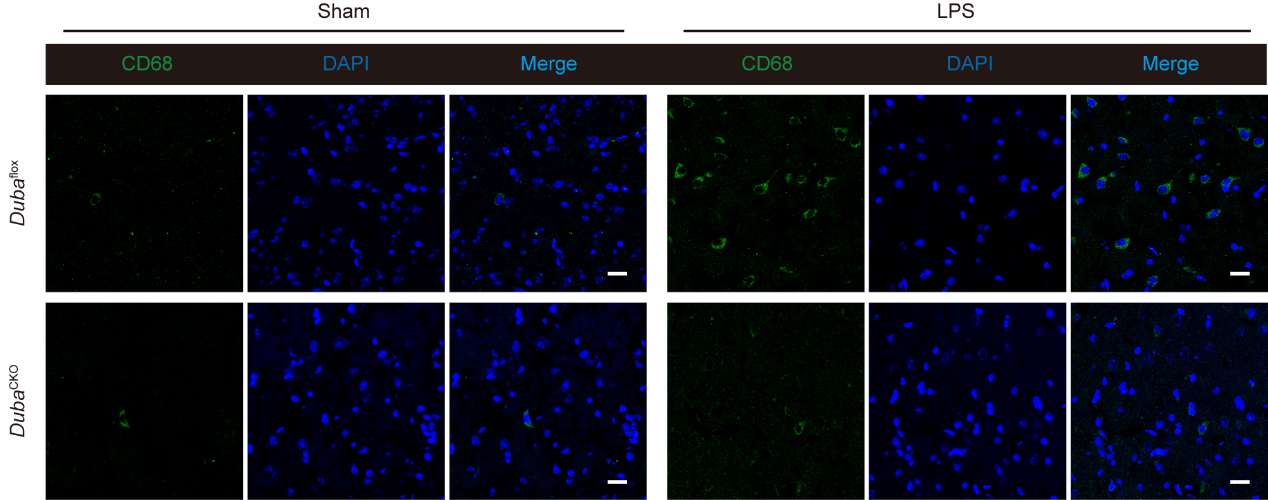


**Supplementary Figure 9. DUBA deficiency reduces the activation of microglia after LPS stimulation.**

Representative CD68 immunofluorescence staining in the ischemic penumbra of *Duba*^flox^ and *Duba*^cko^ mice intraperitoneally injected with LPS (0.5 mg/kg) daily for 7 consecutive days. Scale bar, 20 μm.

**Fig. S10**


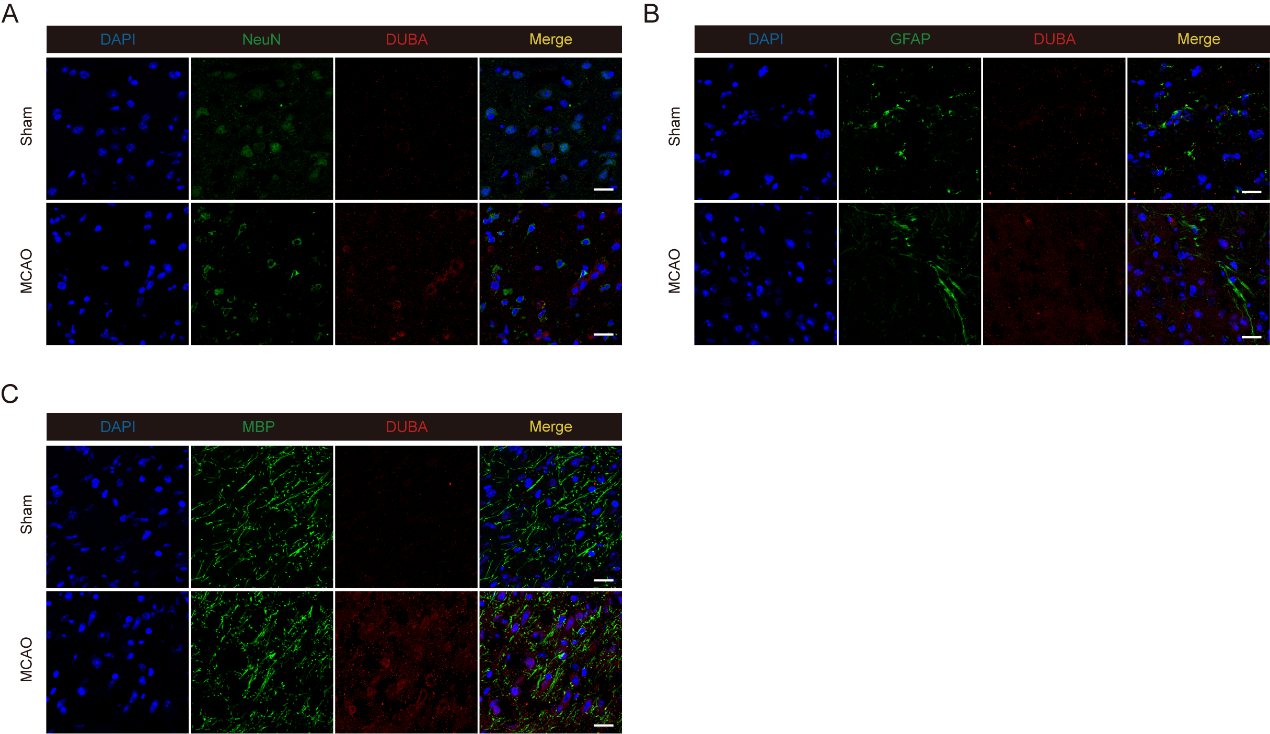


**Supplementary Figure 10. DUBA is not upregulated in neurons, astrocytes, and oligodendrocytes after MCAO.**

(A) Representative immunofluorescence staining of DUBA (red), NeuN (green), and DAPI (blue) in the ischemic penumbra at day 3 after MCAO. Scale bar, 20 μm. (B) Representative immunofluorescence staining of DUBA (red), GFAP (green), and DAPI (blue) in the ischemic penumbra at day 3 after MCAO. Scale bar, 20 μm. (C) Representative immunofluorescence staining of DUBA (red), MBP (green), and DAPI (blue) in the ischemic penumbra at day 3 after MCAO. Scale bar, 20 μm.

**Fig. S11**


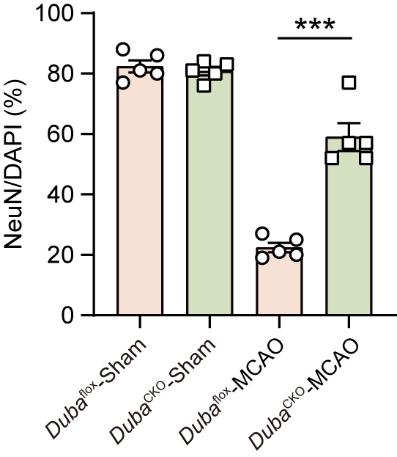


**Supplementary Figure 11. Quantification results for Figure 8C.**

Quantification results for Figure 8C (n = 5). Mean ± SEM. *** *P* < 0.001.

**Fig. S12**


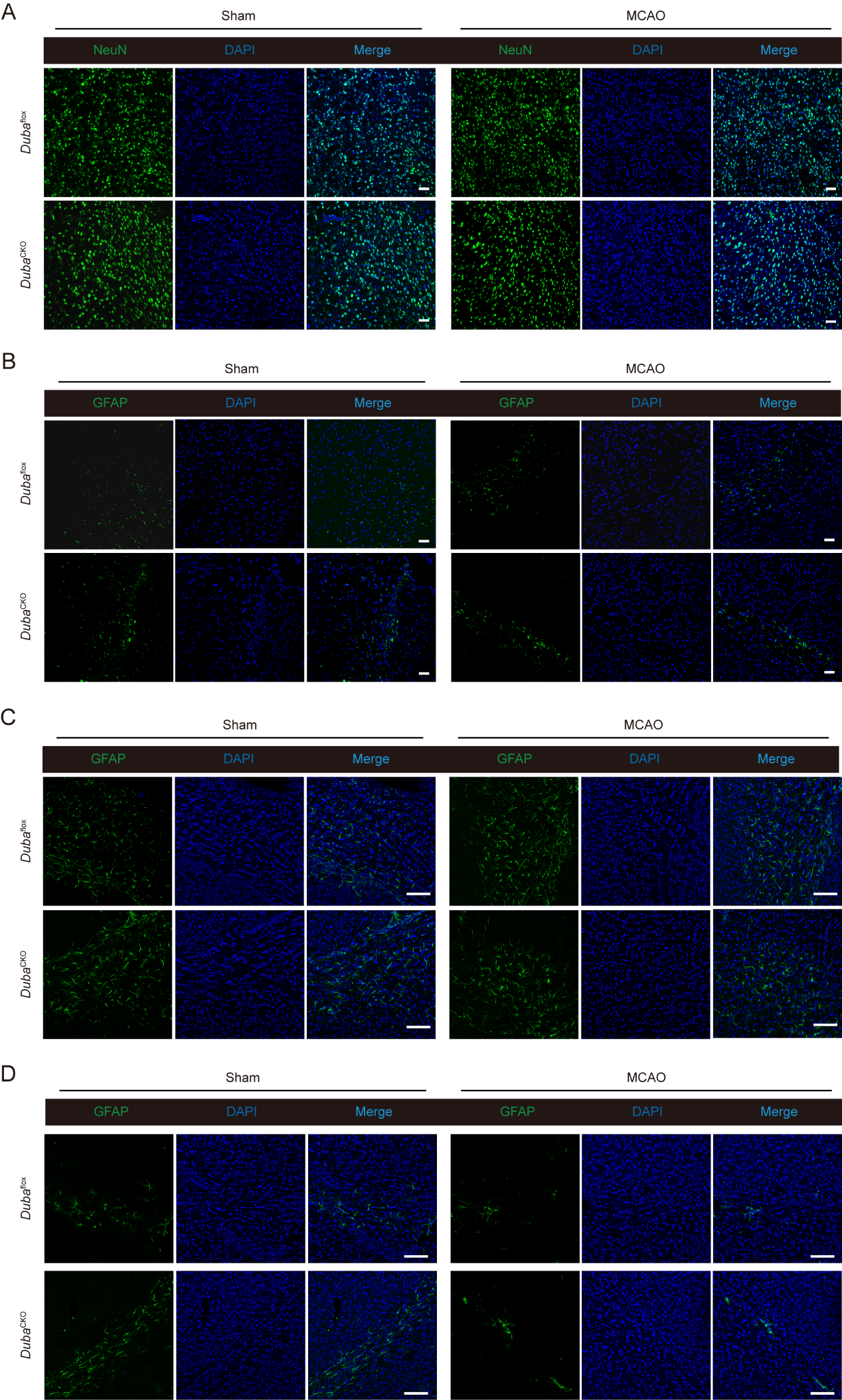


**Supplementary Figure 12. DUBA deletion does not change the number of neurons early after MCAO and the activation of astrocytes.**

(A-B) Representative immunofluorescence staining of NeuN (A) and GFAP (B) in the ischemic penumbra of *Duba*^flox^ and *Duba*^cko^ mice at 6 h after operation. Scale bar, 50 μm. (C-D) Representative immunofluorescence staining of GFAP in the ischemic penumbra (C) and infarct core (D) of *Duba*^flox^ and *Duba*^cko^ mice at 72 h after operation. Scale bar, 100 μm.

**Fig. S13**


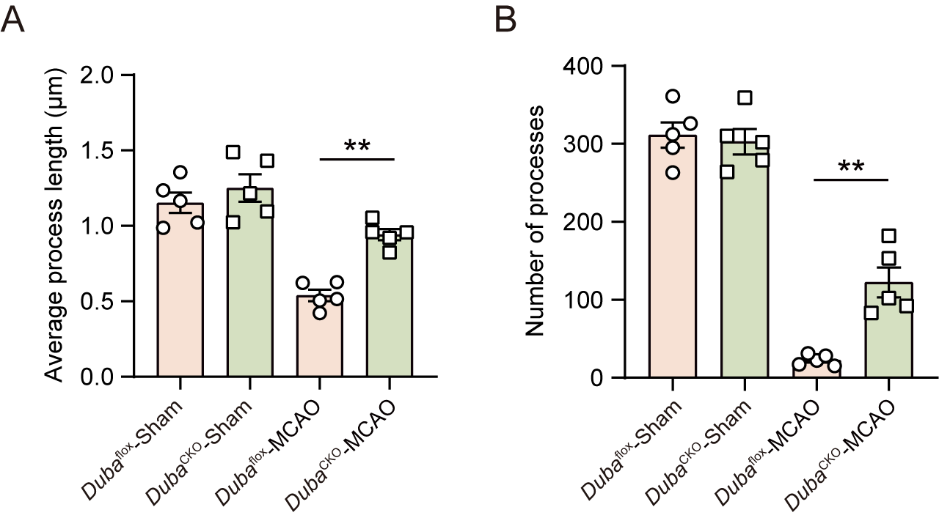


**Supplementary Figure 13. Quantification results for Figure 8I.**

(A-B) Quantification results for average process length (A) and process number (B) of microglia studied in Figure 8I (n = 5). Mean ± SEM. ** *P* < 0.01.

**Fig. S14**


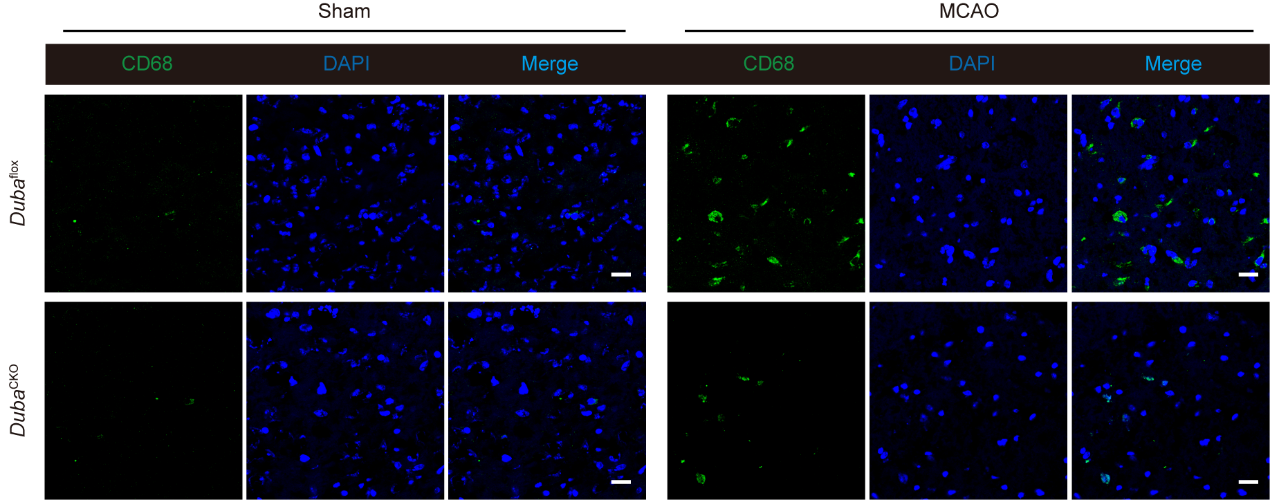


**Supplementary Figure 14. DUBA deficiency reduces the activation of microglia after MCAO.**

Representative CD68 immunofluorescence staining in the ischemic penumbra of *Duba*^flox^ and *Duba*^cko^ mice at day 3 after operation. Scale bar, 20 μm.

**Fig. S15**

**
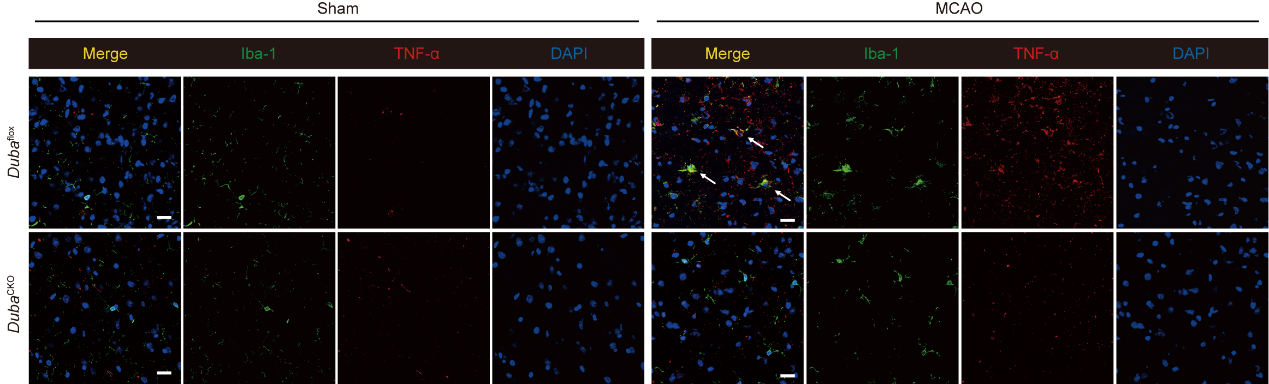
**

**Supplementary Figure 15. DUBA deficiency reduces TNF-α production in microglia after MCAO.**

Seventy-two hours after operation, the ischemic penumbra of *Duba*^flox^ and *Duba*^cko^ mice was analyzed by immunofluorescence with indicated antibodies. Scale bar, 20 μm.

| Supplementary Table 1. Antibody information. |  |  |
| --- | --- | --- |
| Antibody | Source | CAT# |
| Mouse anti-A20/TNFAIP3 (A-12) | Santa Cruz | sc-166692 |
| Mouse anti-IRAK-1 | Santa Cruz | sc-5288 |
| Rabbit mAb anti-OTUD5 (D8Y2U) | Cell Signaling | 20087S |
| Rabbit mAb anti-Iba1/AIF-1 (E4O4W) | Cell Signaling | 17198S |
| Rabbit mAb anti-IKKα (D3W6N) | Cell Signaling | 61294S |
| Rabbit mAb anti-IKKβ (D30C6) | Cell Signaling | 8943S |
| Rabbit mAb anti-IκBα (44D4) | Cell Signaling | 4812S |
| Rabbit mAb anti-p38 MAPK (D13E1) | Cell Signaling | 8690S |
| Rabbit mAb anti-p44/42 MAPK (Erk1/2) (137F5) | Cell Signaling | 4695S |
| Rabbit mAb anti-SAPK/JNK | Cell Signaling | 9252S |
| Rabbit mAb anti-MyD88 (D80F5) | Cell Signaling | 4283S |
| Rabbit mAb anti-NF-κB p65 (D14E12) | Cell Signaling | 8242S |
| Rabbit mAb anti-Phospho-IKKα/β (Ser176/180) (16A6) | Cell Signaling | 2697S |
| Rabbit mAb anti-Phospho-IκBα (Ser32) (14D4) | Cell Signaling | 2859S |
| Rabbit mAb anti-Phospho-p44/42 MAPK (Erk1/2) (Thr202/Tyr204) (D13.14.4E) | Cell Signaling | 4370S |
| Rabbit mAb anti-Phospho-SAPK/JNK (Thr183/Tyr185) (81E11) | Cell Signaling | 4668S |
| Rabbit mAb anti-Phospho-p38 MAPK (Thr180/Tyr182) | Cell Signaling | 9211S |
| Rabbit mAb anti-Phospho-NF-κB p65 (Ser536) (93H1) | Cell Signaling | 3033S |
| Rabbit mAb anti-Phospho-TAK1 (Thr184/187) (90C7) | Cell Signaling | 4508S |
| Rabbit mAb anti-TAK1 (D94D7) | Cell Signaling | 5206S |
| Rabbit mAb anti-Toll-like Receptor 4 (D8L5W) | Cell Signaling | 14358S |
| K48-linkage Specific Polyubiquitin Antibody | Cell Signaling | 4289S |
| Anti-DUBA | Abcam | ab225558 |
| Anti-Iba1 antibody [EPR16589]-Rat IgG2a | Abcam | ab283346 |
| Anti-NeuN antibody [EPR12763] | Abcam | ab177487 |
| Rabbit pAb anti-FLAG | Proteintech | 20543-1-AP |
| Rabbit pAb anti-HA | Proteintech | 51064-2-AP |
| Rabbit pAb anti-MYC | Proteintech | 16286-1-AP |
| Rabbit pAb anti-GFAP | Proteintech | 16825-1-AP |
| Rabbit pAb anti-IRAK1 | Proteintech | 10478-2-AP |
| Mouse mAb anti-TNF Alpha | Proteintech | 60291-1-Ig |
| Mouse anti Iba1 | Sigma Aldrich | SAB2702364 |
| GAPDH antibody | Bioworld | MB001 |
| HRP-labeled Goat Anti-Mouse IgG (H+L) | Beyotime | A0216 |
| HRP-labeled Goat Anti-Rabbit IgG (H+L) | Beyotime | A0208 |

**Supplementary Table 2. Primers for qRT-PCR.**

| Gene | Species | Forward primer (5' - 3') | Reverse primer (5' - 3') |
| --- | --- | --- | --- |
| *Il1b* | mouse | GAAATGCCACCTTTTGACAGTG | TGGATGCTCTCATCAGGACAG |
| *Il6* | mouse | CTCCCAACAGACCTGTCTATAC | CCATTGCACAACTCTTTTCTCA |
| *Tnf* | mouse | CAGGCGGTGCCTATGTCTC | CGATCACCCCGAAGTTCAGTAG |
| *Cxcl10* | mouse | CCAAGTGCTGCCGTCATTTTC | GGCTCGCAGGGATGATTTCAA |
| *Ccl2* | mouse | TAAAAACCTGGATCGGAACCAAA | GCATTAGCTTCAGATTTACGGGT |
| *Nos2* | mouse | GTTCTCAGCCCAACAATACAAGA | GTGGACGGGTCGATGTCAC |
| *Actb* | mouse | GTGACGTTGACATCCGTAAAGA | GCCGGACTCATCGTACTCC |
| *Duba* | mouse | GCCACTGTAGAACAGCAGGA | AGCCCGAAATAGACAGGCAC |
| *Irak1* | mouse | CAGAGGCAAAACTCCCAACA | GCAGCAGCCCTTTACCACTTA |
